# Individual Mechanical Energy Expenditure Regimens Vary Seasonally with Weather, Sex, Age and Body Condition in a Generalist Carnivore Population: Support for Inter-Individual Tactical Diversity

**DOI:** 10.3390/ani15111560

**Published:** 2025-05-27

**Authors:** Julius G. Bright Ross, Andrew Markham, Michael J. Noonan, Christina D. Buesching, Erin Connolly, Denise W. Pallett, Yadvinder Malhi, David W. Macdonald, Chris Newman

**Affiliations:** 1 Wildlife Conservation Research Unit, Department of Biology, University of Oxford, The Recanati-Kaplan Centre, Tubney House, Abingdon Rd, Tubney OX13 5QL, UK; jbrightross@gmail.com (J.G.B.R.); david.macdonald@biology.ox.ac.uk (D.W.M.); 2 Department of Computer Science, University of Oxford, Wolfson Building, Parks Road, Oxford OX2 3QD, UK; andrew.markham@cs.ox.ac.uk; 3 The Irving K. Barber School of Arts and Sciences, The University of British Columbia, Okanagan Campus, Kelowna, BC V1V 1V7, Canada; michael.noonan@ubc.ca (M.J.N.); christina.buesching@ubc.ca (C.D.B.); 4 Centre for Biodiversity and Environment Research, Department of Genetics, Evolution and Environment, University College London, London WC1E 6BT, UK; erin.connolly.22@ucl.ac.uk; 5 UK Centre for Ecology & Hydrology, Wallingford OX10 8BB, UK; dwpa@ceh.ac.uk; 6 Environmental Change Institute, School of Geography and the Environment, University of Oxford, South Parks Road, Oxford OX1 3QY, UK; yadvinder.malhi@ouce.ox.ac.uk; 7 Carnivore Ecology and Conservation Research Group, Institute of Agriculture, Tokyo University of Agriculture and Technology, Saiwaicho 3-5-8, Fuchu, Tokyo 183-8509, Japan; 8 Department for Continuing Education, University of Oxford, Rewley House, 1 Wellington Square, Oxford OX1 2JA, UK

**Keywords:** activity profiles, age differences, behavioral plasticity, behavioral thermoregulation, dynamic heterogeneity, tri-axial accelerometry

## Abstract

Diverse individual energy-budgeting tactics within wild populations provide resilience to natural fluctuations in food availability and expenditure costs. Although substantial heterogeneity in activity-related energy expenditure has been documented, few studies differentiate between responses to the environment and inter-individual differences stemming from life history, allometry, or somatic stores. Using tri-axial accelerometry, complemented by diet analysis, we investigated inter-individual within-season variation in overall dynamic body acceleration (ODBA; activity intensity measure) and “Activity” (above an ODBA threshold) in a high-density population of European badgers (*Meles meles*). Weather (including wind speed) affected ODBA and activity according to predictors of earthworm (food) availability and cooling potential. In spring, maximal ODBA expenditure at intermediate rainfall and temperature values suggested that badgers traded foraging success against thermoregulatory losses, where lower-condition badgers maintained higher spring ODBA irrespective of temperature while badgers in better body condition reduced ODBA at colder temperatures. Conversely, in summer, lower-condition badgers modulated ODBA according to temperature, likely in response to super-abundant food supply. Between 35% (spring, summer) and 57% (autumn) of residual total daily ODBA variance related to inter-individual differences unexplained by seasonal predictors, suggesting within-season tactical activity typologies. We propose that this heterogeneity among individual energy-expenditure profiles may contribute to population resilience under rapid environmental change.

## Simple Summary

Although animals are typically modeled as making uniform responses to weather conditions in the context of climate change, in reality the resilience and adaptive capacity of a population or species relate to differences in the way individuals respond. Using European badgers (*Meles meles*) in a high-density population as a model, we investigated how overall dynamic body acceleration (ODBA, a measure of activity intensity) and “Activity” (above an ODBA threshold) differed between individuals across seasons. Weather (including wind speed) affected badger ODBA and activity according to predictors of food resource (earthworm) availability and potential for cooling effects. In spring, maximal ODBA was expended with intermediate rainfall and temperatures, suggesting that badgers traded off foraging success against thermoregulatory losses. Crucially, ODBA plasticity to temperature was highly dependent on individual body condition. Thinner badgers maintained high spring ODBA irrespective of temperature, while fatter badgers reduced ODBA at colder temperatures. Conversely, in summer, thin badgers modulated ODBA according to temperature, likely in response to super-abundant food supply. Ultimately, 35% to 57% of remaining variation in daily ODBA was due to inter-individual activity profiles beyond the effects tested. We conclude that heterogeneity among individual energy expenditure profiles may contribute to population resilience under rapid environmental change.

## 1. Introduction

Vulnerability to human-induced rapid environmental change (HIREC) [1] generally focuses on the species as the minimal unit of investigation [2,3], yet, fundamentally, natural selection operates on the individual [4,5]. Energy provides a common currency [6] for quantifying how individuals within the same population balance obligate and elective activities. Overarching constraints at the species level determine the context within which this balancing occurs; such as diet diversity [7], average body size [8], climatic constraints in achieving thermoneutrality [9], reproductive strategies [10], and predator pressures [11]. Ultimately, however, in order to optimize energy allocation between survival and reproduction, and to have sufficiently predictable energy security to plan tactical energy budgets [12,13], each individual aims to balance available energy inputs (food supply) with outputs (physiological homeostasis and activity) [14] according to circumstances (sex, age, body-condition, social status, etc.).

Under stable and consistent environmental conditions, directional selection should drive energy budgeting canalization [15] after accounting for variation stemming from intrinsic effects such as sex [16], age [17], and body condition [12]. This would result in a narrow range of optimized tactics prevailing across the entire population or a small set of alternating strategies under density-dependent fluctuating selection [18]. In reality, environments are seldom so predictable [19], and multiple pathways to equivalent fitness outcomes can produce tactical heterogeneity between individuals at both fine (daily) [20] and coarse (seasonal) [21] timeframes. This can result in disruptive selection, giving individuals with widely differing behavioral tactics—and consequently, energy expenditure—a stochastic fitness advantage over more moderate tacticians [22]; or, alternatively, fluctuating selection for tactics that suit alternating environments [18]. In either case, inter-individual tactical diversity provides a population with resilience to change, where some individuals will exhibit plastic variation in energy budgeting that may make them “pre-adapted” to newly emergent conditions [23,24].

Rapid anthropogenic climate change reduces environmental predictability and destabilizes the selection pressures that govern energy allocation tactics [13,25]. These effects are compounded by other forms of HIREC disturbances, such as habitat loss [26], such that even vagile organisms may be unable to fully track their optimal bioclimatic niche [27,28]. Coping strategies in situ are therefore often essential for population and species persistence [29,30], but the short timescales involved limit the response effectiveness of genetic adaptation [19,31,32]. Plastic changes in energy expenditure, therefore, represent an essential coping strategy [33,34], although little attention has been given to how activity budgets, as the most pliable component of energy expenditure modification, are simultaneously (a) constrained between individuals experiencing the same contemporary environmental conditions (e.g., site, weather, predators, etc.) after accounting for sex, age, etc.; (b) limited by intra-individual consistency, potentially reflecting tactical activity budgeting types; and, crucially, (c) affected by inter-individual differences in responses to the shared environmental conditions they experience (differential plasticity) [35].

Energy intake and its environmental covariates are frequently characterized through diet analysis [36], but quantifying how individuals deploy the energy assimilated from their diet is more challenging. Recently, advances in bi- and tri-axial accelerometry have enabled the intensity of activity to be quantified through overall dynamic body acceleration (ODBA) [37]. ODBA correlates with total metabolism in a wide range of species [38,39], particularly at short timescales [40], although the ability of ODBA to predict metabolic rate declines as the contribution of non-movement-related factors, such as heat production, rises [41]. Nevertheless, ODBA generally provides a robust proxy for movement-based power [41], making it a good proxy for mechanical energy expenditure [42].

Here, we used collar-mounted custom tri-axial accelerometers to examine variability in individual activity regimens in a population of wild European badgers (*Meles meles*, hereafter “badger”). Badgers provide a highly informative model system for studying individual activity, exhibiting strongly weather-dependent foraging patterns according to prevailing conditions and food availability [43,44,45]. At our high-density population study site, badgers live in social groups averaging 6.8 individuals (range 1–28), comprising an overall population of est. 248 individuals in 2016 [46]. Moreover, badger groups occupy subterranean dens (setts), providing weather refugia [47] from being above ground in suboptimal thermal conditions [48]—particularly for fatter badgers with lower motivation to forage [49]. On fine timescales, the availability of earthworms (particularly *Lumbricus terrestris*), a primary though not obligate food item consumed in our study region [50], also varies extensively with weather conditions [51]. Although badgers mate post-partum from mid-February following winter torpor [52,53], blastocyst implantation is delayed until the onset of the following winter [54], which enables badgers to align the energetically costly stages of mating with peak spring food abundance [50,55], focusing the majority of these costly reproductive investments into one season. During the transition from summer to autumn, badgers undergo substantial seasonal increases in body fat reserves to prepare for winter torpor [25].

To investigate how weather affected daily activity in badgers (quantified at the hourly scale), we tested how “Activity” (binary thresholded using ODBA values—denoted with a capital “A” to differentiate from general activity) and absolute “ODBA” (the intensity of activity) were linked to prevailing weather conditions. Rainfall and temperature can affect wild animals’ survival probabilities non-linearly, where intermediate conditions are often optimal [30,56]; therefore, we also tested these two covariates for quadratic effects. Furthermore, we examined whether under-evaluated weather covariates (wind speed, relative humidity, and a range of soil conditions) can influence activity and ODBA; for instance, wind speed reduces earthworm surfacing rates and interferes with predator sensory perception [57], as well as affecting convective heat loss [58]. For background seasonal energy intake context, we also conducted a year-long fecal dietary analysis.

Within the context of environmental conditions—and seasonal relevance—we quantified how activity budgeting varied in relation to intrinsic traits (sex, age, and body condition). Particularly, we predicted that older badgers might exhibit risk-averse activity patterns due to geriatric frailty [25]. We then examined whether there was substantial inter-individual tactical response heterogeneity in the population, and whether individuals exhibited consistent energy expenditure typologies across measurements (i.e., “energetic” vs. “lethargic” activity types).

## 2. Materials and Methods

### 2.1. Data Collection

Badger data were collected as part of a long-term demographic study in Wytham Woods (Oxfordshire, UK 51°46′ N, 1°20′ W) [59]. In May (“spring”), September (“summer”), and November (“autumn”) of 2018 and 2019, we captured badgers at setts used by three different social groups (n = 75, see Table 1 below for seasonal sample sizes) and transported them to a central field station. We sedated badgers with 0.2 mL ketamine hydrochloride (Vetalar, Zoetis UK Limited, Leatherhead, UK) per kg of body weight via intramuscular injection [60] and equipped the first 9–12 adults captured each season with custom-built tri-axial accelerometers (SensorTile turn-key sensor modules, STEVAL-STLK01V1, STMicroelectronics, Geneve, Switzerland) within 3D-printed ABS plastic casings attached to padded dog collars (averaging 1.34% of bodyweight). In the collared subset, age was inferred from the date of the individual’s initial capture as a cub; only one badger was first captured as an adult, and its age was estimated from tooth wear [46]. Only adults were collared, due to welfare considerations for growing cubs. After sedation, we measured each individual’s weight (to the nearest 0.1 kg) and body length (to the nearest 5 mm), then computed a body-condition index (BCI) as a measure of body fat reserves [25,61]:

(1) $$BCI=\frac{{log}_{e}(mass)}{{log}_{e}(length)}$$

Female breeding success could not be deduced unequivocally, and so was not tested explicitly as an explanatory variable; however, we used teat length and width and assessed recent lactation status visually to establish whether a female had lactated in the year of capture [25,62]. Badgers were released at their place of capture in the afternoon of the same day.

These badgers were then targeted for recapture 6–10 days later to recover collars (see Table 1 for a breakdown of collar recovery rates). In four cases, individuals could not be recaptured within seasonal trapping sessions (12 days) and collars were recovered at later trappings. A longer deployment was made from 10 September 2018. Catastrophic data failure occurred from a hardware incompatibility test in November 2018, resulting in only one year’s data for autumn deployments.

All captures were licensed under the Badger Act (1992) (Natural England license 2019-2020-4417) and the Animals (Scientific Procedures) Act (1986) (PPL 30/3379).

We obtained hourly weather data from the UK Environmental Change Network (ECN, terrestrial site T08) meteorological station at Wytham Woods [63]. We used: (a) mean air temperature (“Temp”, °C); (b) total rainfall (“Rain”, mm); (c) wind speed (“Wind”, m/S); (d) mean relative humidity (“RH”, in %); (e) soil moisture at 20 cm (“SoilM”, %); and (f) soil temperature at 10 and 30 cm (“SoilT_10_” and “SoilT_30_”, °C). During September 2019 deployments, the rainfall gauge was obstructed. We cross-referenced these days with data from the Radcliffe Meteorological Station 6 km away in Oxford and established that total daily precipitation for 7 of these 9 days was 0 mm or only trace amounts; therefore, we substituted hourly precipitation data with 0s for these days, removing the two days for which we could not determine hourly data. We then compared each weather metric during the accelerometry data periods to the long-term average for these same dates (1991–2015). Although various covariates differed significantly from the long-term mean (Supplementary S1), most deviations were within one standard deviation of these means, making these periods broadly representative of typical seasonal values (Table S1), with two exceptions: SoilT_10_, which was 1.0 standard deviation higher than the long-term average in spring 2018 (*p* < 0.001), and SoilM, which was 1.3 standard deviations lower than the long-term average in spring 2019 (*p* < 0.001), indicating unusually warm and dry soil conditions. We found substantial seasonal correlations between various weather metrics (Supplementary S1), and so only evaluated models without collinear subsets of predictors in Section 2.3, Section 2.4 and Section 2.5.

As dietary context for seasonal energy availability, we analyzed feces from Apr-Nov 2018. While seasonal diet patterns can vary year-to-year, this variation was not the analytical subject of this study and therefore we considered a single year’s dietary data to be broadly representative of seasonal patterns for both years. We counted earthworm chaetae present in effluent washed through sieved scats, producing a measure of average chaetae found in 10 1-cm^2^ squares on a Petri dish [64,65]. We also calculated the monthly frequency of occurrence (FO = occurrences/samples) and relative frequency of occurrence (RFO = occurrences/total diet item occurrences) for each diet item (Supplementary S4).

### 2.2. Processing Accelerometry Data

We quantified mechanical energy expenditure from overall dynamic body acceleration (ODBA) as the L1-norm of the three acceleration channels [42,66]:

*ODBA* = |*A_x_*| + |*A_y_*| + |*A_z_*| (2)

A_{x,y,z}_ represent the difference between the mean and the midpoint of each channel over a 2 s window, divided by 2^14^, the g-force programmed for these accelerometers.

We used the sum of hourly ODBA as a measure of hourly mechanical energy expenditure and used minute-averaged ODBA (see Supplementary S2) to calculate activity at both the hourly and daily scales. We used a sensitivity analysis [67] to delimit an active/inactive threshold for minute-averaged ODBA (0.28, Supplementary S2) and used it to compute (a) hourly activity (binary: active defined as above threshold for >30 min in the hour) and (b) the total number of active minutes (above the same threshold) per night (noon-to-noon), to characterize daily activity duration.

We detected significantly lower activity during the first day after release compared to subsequent days [68], and so used data only from the second day of deployment onwards in our models. We also excluded data from the last day of each deployment, as the exact time of capture during the last night of activity was uncertain. Finally, we excluded one badger with a naturally occurring limp from spring 2018, which expended only 73% of the population’s average ODBA. This resulted in 6238 “badger-hours” of data (spring: 1933 h/14 deployments/12 individuals; summer: 3585 h/19 deployments/16 individuals; autumn: 720 h/7 deployments/7 individuals).

### 2.3. Seasonal Drivers of ODBA and Activity

For each season, we constructed a full generalized additive mixed model (GAMM, *mgcv* package) [69] to describe ODBA expenditure, including ODBA at the hourly scale (log_2_-transformed; higher values had greater variance than lower values—Supplementary S2), as badger activity follows a clear circadian pattern and can respond to environmental covariates at a finer scale than an entire night. For interpretation, we then summed hourly model predictions to infer the total daily ODBA per individual under given conditions. Therefore, we modeled hourly ODBA seasonally as a function of: (a) a cyclic cubic regression spline of hour of day; (b) weather covariates, including quadratic terms for rainfall and temperature; (c) individual intrinsic covariates (age, sex, and BCI); (d) pairwise BCI interactions with sex, temperature, and rainfall; and (e) random intercepts for individuals. We standardized all continuous covariates to a mean of 0 and a standard deviation of 1 (*standardize* package) [70]. Autumn data included only two female badgers (from seven), so we did not include sex as a covariate in that model.

We verified that random intercepts improved model fit for the full model structure (after Zuur et al., ΔAIC > 10 in all seasons) [71]. We detected substantial autocorrelation in the residuals, and so we included an AR-1 autoregressive term in each seasonal model. We then performed all-subsets model selection for each season using Akaike’s Information Criterion (AIC, *MuMIn* package) [72], excluding models with collinear covariates (Supplementary S1). We also performed equivalent selection for models constructed without Supplementary terms and on thinned data to evaluate the effects of autocorrelation on term selection. We proceeded for each season with the model exhibiting the lowest AIC with AR-1 terms (see Supplementary S3 for models with ΔAIC < 2 and full coefficients for top models from all three model selection approaches, which produced similar results).

We used the same procedure to select models predicting hourly activity (binomial, 1/0) for each season. Due to the computational intensity of evaluating binomial models with autoregressive terms, we performed activity model selection without modeling residual autocorrelation (see Supplementary S3 for models with ΔAIC < 2). We then included AR-1 terms in the selected seasonal models.

### 2.4. Inter-Individual Environmental Response Heterogeneity

We investigated inter-individual differences in ODBA expenditure per night in two ways: (i) in seasonal hourly ODBA and activity models, we included interactions between individual traits (age, sex, and BCI) and key environmental metrics (see Section 2.1) to establish potential drivers of inter-individual plasticity; (ii) remaining unexplained plasticity was investigated by re-computing the best seasonal model of hourly ODBA with random individual slopes for weather metrics, limited to the period between sunset and sunrise. We used AIC to compare separate models with and without random slopes for each weather covariate.

### 2.5. Intra-Individual Consistency (Activity Types)

Variance in hourly ODBA relating to the day/night cycle exceeded inter-individual variance; therefore, the random intercepts from hourly ODBA models could not sufficiently quantify individual activity types. Instead, we used intra-class correlation (ICC) coefficients from seasonal models of total nightly ODBA (sum of hourly ODBA values from noon-to-noon) as a function of the covariates selected in each seasonal hourly ODBA model, in which hourly weather covariates were averaged between sunset and sunrise (derived from the *suncalc* package) [73]. We performed an identical procedure with models of the total minutes each individual was active per night. We calculated ICC as the ratio of variance described by individual intercepts to total variance, after Nakagawa and Schielzeth [74]:

(3) $$ICC=\frac{var(Individual)}{var(Individual+Residual)}$$

## 3. Results

### 3.1. Individual and Seasonal Variation in ODBA and Activity Patterns

**Figure 1:**
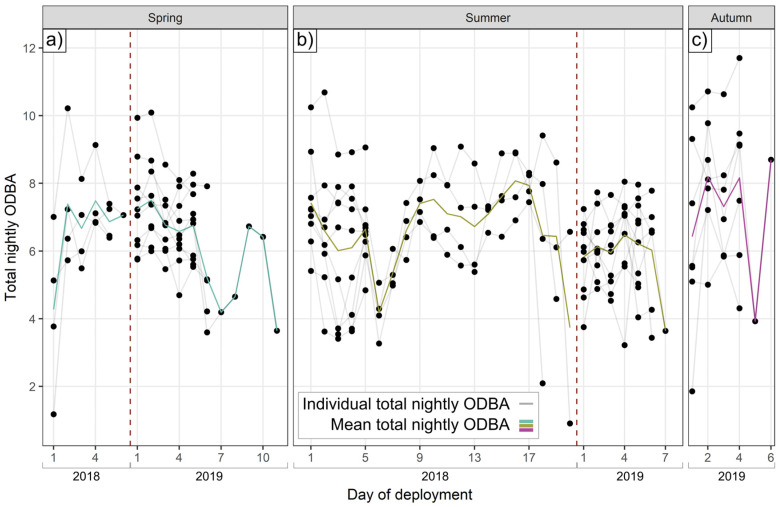
Nightly ODBA variation. Variation in total nightly ODBA expended by individual badgers (connected by gray lines) under the same environmental conditions in (**a**) spring, (**b**) summer, and (**c**) autumn. Colored lines show averages of nightly ODBA, and vertical dotted red lines separate datasets by year.

Individuals exhibited substantially different total ODBA on the same nights, under the same conditions (see Figure 1). On average, the most active badger on a given night expended 1.9 times (range 1.1–5.9x) the ODBA of the least active.

**Figure 2:**
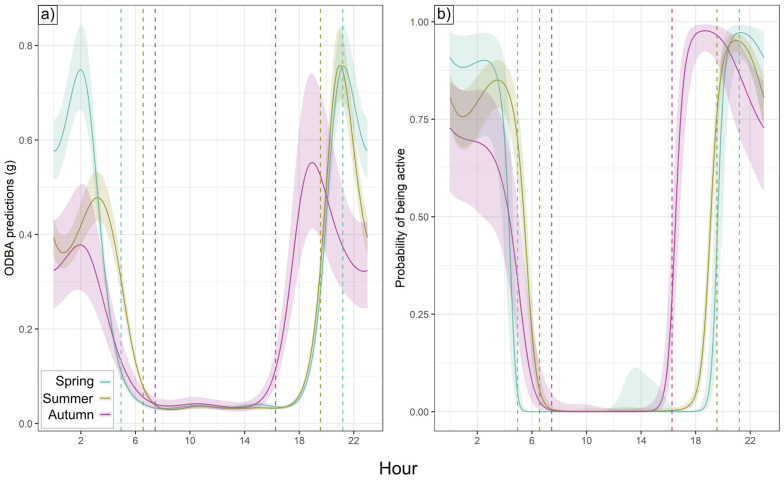
Hourly trends by season. Output predictions from smooth terms (cyclic cubic spline) in (**a**) ODBA models and (**b**) activity models by season as a function of hour of day. Shaded region shows 95% confidence interval for hourly relationship; vertical dotted lines show average sunrise and sunset for deployment periods (blue = spring, green = summer, purple = autumn).

Nightly badger activity duration was longest in autumn (569.8 ± 146.4 SD minutes), shortest in spring (502.3 ± 109.1 min), and intermediate in summer (526.8 ± 110.0 min). Total nightly ODBA was, however, nearly identical in spring and summer (6.54 ± 1.58 and 6.51 ± 1.48, respectively) and somewhat higher in autumn (7.44 ± 2.29). Nightly ODBA schedules (from hourly ODBA models) exhibited one peak around sunset and one before sunrise (Figure 2a). Activity patterns were similar (Figure 2b), although in autumn, the activity exhibited only a post-sunset peak, with no elevated probability of pre-sunrise activity. While individuals were more likely than not to be active on any given night (Figure 2b), 12.1% (spring), 20.9% (summer), and 27.3% (autumn) of all night-time badger hours (between ODBA peaks) were below the activity threshold, representing substantial episodes of nocturnal inactivity across all seasons. Diurnal activity did not exhibit this variability, with only 2 h of diurnal activity across all badgers (09:00–15:00), both during summer. While post-sunset ODBA/activity peaks occurred 1.5–2.5 h after sunset in summer and autumn, the spring peak coincided with sunset. Spring and summer post-sunset ODBA peaks were 36.9% and 37.2% higher than in autumn, respectively, while the pre-sunrise activity peak was only ODBA-intensive in spring, being 56.6% and 98.0% higher than in summer and autumn, respectively (Figure 2a). There were fewer seasonal differences between peaks in the hourly activity relationship, where >95% of badgers in all seasons were active during the sunset peak; in spring and summer, 90.9% and 85.1% of badgers, respectively, were active during the pre-sunrise peak, dropping to 66.4% at 03:00 (pre-inactivity; no second activity peak) in autumn (Figure 2b).

Model fit was high for hourly ODBA in spring (R^2^ = 0.819), slightly lower for summer (R^2^ = 0.718), and lowest in autumn, although still explaining over 50% of variance (R^2^ = 0.581). Model fit was high for all hourly activity models, with 94.6% (spring), 88.4% (summer), and 84.7% (autumn) of hours classified accurately as either active or inactive. Matthews correlation coefficients (MCCs), which measure correlations of classified data points while accounting for sensitivity and specificity (from −1 for perfect negative correlation to +1 for perfect classification), supported the high classification reliability of seasonal models, with MCCs of 0.883 (spring), 0.761 (summer), and 0.701 (autumn). Five-fold cross-validation showed this classification success was robust to data sub-setting, producing accuracies of 94.3% (spring, MCC = 0.877), 88.3% (summer, MCC = 0.759), and 83.5% (autumn, MCC = 0.670).

### 3.2. Seasonal Drivers of Daily ODBA/Activity

#### 3.2.1. Weather Drivers

**Figure 3:**
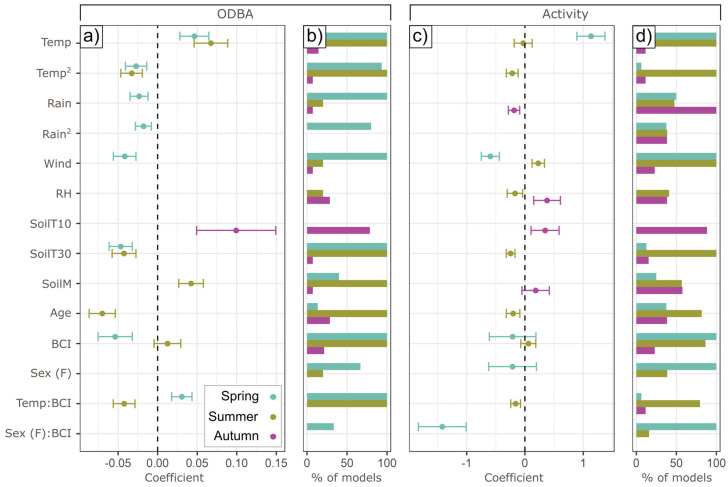
Drivers of ODBA/activity. Panels (**a**,**c**) show the coefficients of lowest-AIC seasonal models for hourly ODBA and activity (±SE); panels (**b**,**d**) show the percentage of models with ΔAIC < 2 from the best model that included the term. Vertical dotted line depicts 0 coefficient value.

Figure 3 summarizes covariate coefficient values and presence in top models.

**Figure 4:**
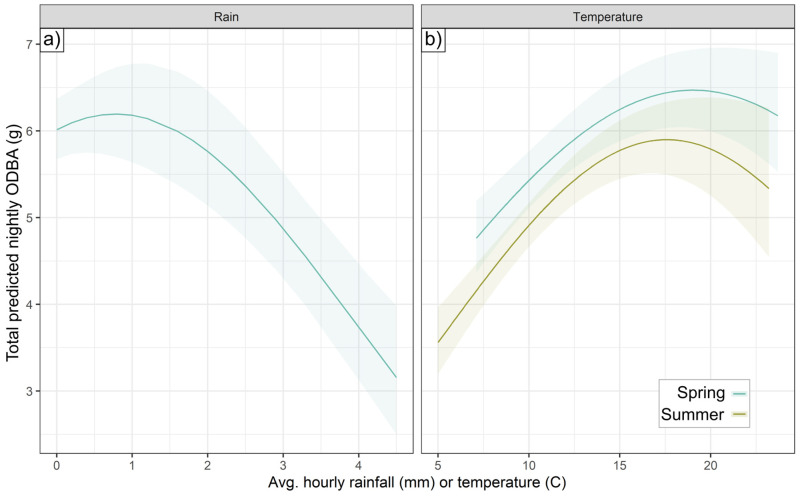
Non-linear effects of temperature and rainfall on nightly ODBA. Total ODBA expended per day (sum of hourly predictions) as a function of (**a**) rainfall or (**b**) temperature.

Temperature affected ODBA quadratically in both spring and summer, with a positive effect of higher temperatures below an optimum, and a negative effect beyond that (Figure 4)—the particular optimum was determined by BCI (see Section 3.3). In spring, higher temperatures were linearly associated with higher activity probability, but summer activity probability peaked at intermediate temperatures (13.1 °C). In spring, rainfall had a similar effect to temperature, with rainfall beyond an optimal point (0.8 mm h^−1^) reducing hourly ODBA. In autumn, higher rainfall correlated negatively with activity, with a 25.6% lower probability of autumnal activity (±SE, 2.2–47.4%) at the highest rainfall values (1 mm h^−1^). There was no effect of rainfall on summer or autumn ODBA, or on spring or summer activity.

Wind speed correlated negatively with both ODBA and activity in spring. The highest speeds (3.8 m s^−1^) coincided with a 24.2% ODBA reduction (12.1–34.7% SE) compared to the lowest speeds (0.3 m s^−1^) but correlated positively with summer activity (predicting up to 19.4% greater likelihood of activity at highest wind speeds—6.8 m s^−1^, SE 9.2–28.7%). Although RH had a positive effect on activity in autumn and a negative effect in summer, these effects lost statistical significance (*p* = 0.10 and 0.20, respectively) after accounting for autocorrelation, and RH was not retained in the best ODBA model for any season.

SoilT_10_ was only retained in autumn models (Temp and SoilT_10_ were collinear, and Temp was consistently selected over SoilT_10_ in other models, Figure 3b,d), with ODBA and activity substantially higher at the highest soil temperatures (up to 44.3% higher activity probability, 9.2–71.6% SE, with total ODBA expended being 2.3-fold higher, 1.4–3.9x SE). SoilT_30_, conversely, had a negative association with ODBA in both spring and summer (23.1%, 11.5–33.2% SE and 28.1, 15.6–38.8% SE reduction in nightly expenditure, respectively), and with activity in summer. Higher SoilM values were associated with up to a 1.4-fold greater nightly summer ODBA (1.2–1.6x SE). SoilM was also retained in autumn activity models but fell below statistical significance after modeling residual autocorrelation (*p* = 0.43).

#### 3.2.2. Intrinsic Drivers of ODBA and Activity

An individual’s age related to ODBA and activity only in summer, when it correlated strongly with lower ODBA (a 13-year-old badger’s total per diem ODBA was 31.1% lower than a 1-year-old’s, 21.0–39.9% SE) and slightly less strongly with lower activity (only 12.8% reduction for the same contrast, 2.6–23.6% SE).

**Figure 5:**
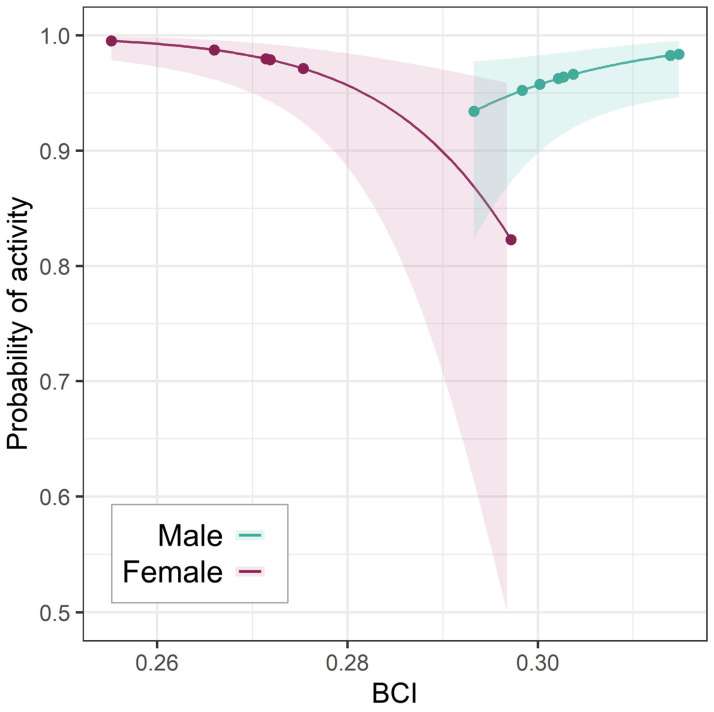
Effect of BCI on spring activity probability. Relationship between BCI and spring activity probability (with 95% CI) for the two sexes; points show the BCI values in the dataset.

Sex was not retained in any ODBA models. However, in spring only, males (positive) and females (negative) had opposite relationships between BCI and activity (Figure 5). Although this negative female effect was in part shaped by one high-BCI female—the only one who had not lactated in the year of collaring—the interaction term remained significant (*p* = 0.01) after re-running the model to exclude that individual.

### 3.3. Inter-Individual Response Variability: Explanatory Factors

**Figure 6:**
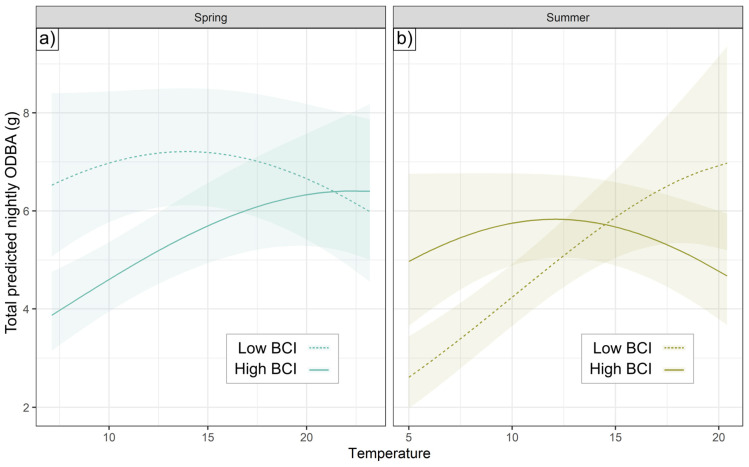
Seasonal BCI-Temp interaction. An individual’s total ODBA in (**a**) spring and (**b**) summer (with 95% CI) varied differently according to temperature for low-BCI (dashed) and high-BCI (solid) individuals. Low and high BCI reflect the most extreme contrasts in our sample: see main text for values relative to population context. *X*-axis spans temperatures experienced by badgers between 17:00 and 7:00 h in seasonal data.

No model with a random slope by an individual for a weather metric had a lower AIC value than the best random-intercept model for each season. A random slope for temperature increased the log-likelihood of models in spring and summer, but not significantly (*p* = 0.28 and 0.18, respectively). However, in spring and summer, individuals differed substantially in their ODBA response to temperature as a function of BCI. In spring, the lowest condition badgers collared (with BCIs in the 0.9th percentile for their social groups) had consistently high nightly ODBA (with predicted values from 6.0 to 7.2), while there was a positive relationship between temperature and ODBA for the higher-condition badgers in our sample (61.3th percentile), ranging from 3.9 to 6.4 (Figure 6a). In summer (Figure 6b), the relationship was inverted, where lower condition badgers (1.2th percentile) responded to Temp, with total ODBA ranging from 2.6 to 7.0, while higher condition badgers (97.4th percentile) remained consistently around 5.6. A similar (but marginally significant, *p* = 0.059) activity relationship occurred for summer but not spring (Figure 3c,d). Neither BCI nor its interactions with extrinsic conditions were retained in autumn models.

### 3.4. Intra-Individual Activity Consistency

After accounting for terms retained in each seasonal ODBA model and for sex, age and BCI, some badgers were consistently active for longer, and expended consistently more ODBA per night, than others.

**Figure 7:**
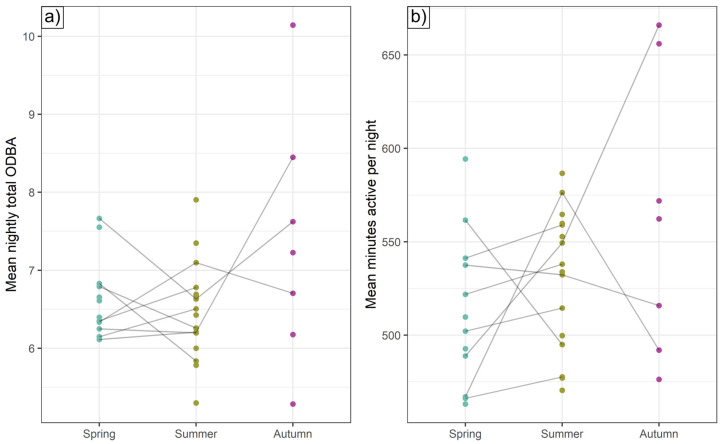
Individual intercepts. Under average seasonal environmental conditions and assuming average age and BCI, the expected (**a**) total nightly ODBA and (**b**) number of nightly minutes spent active for different individuals. Lines connect seasonal estimates for the same individual.

**Figure 8:**
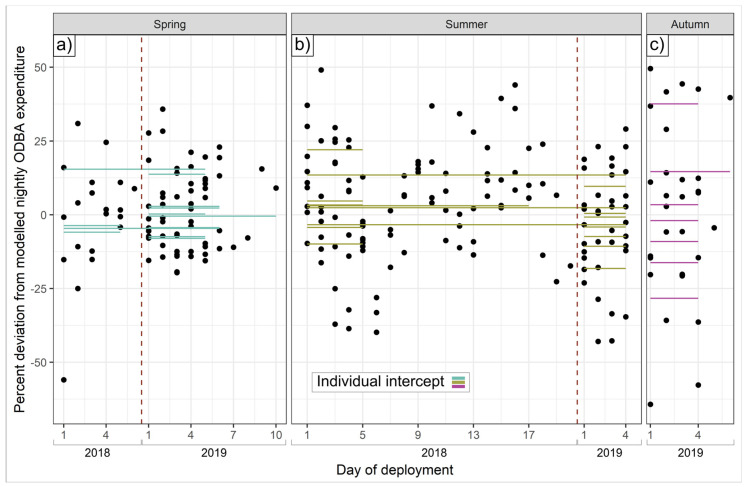
Intra-individual consistency in nightly ODBA deviations. Dots show residual deviations from average predicted nightly ODBA expenditure, in %; horizontal lines show individual intercepts (average deviation from population mean; (**a**) spring, (**b**) summer, (**c**) autumn). Vertical dotted red lines separate datasets by year.

ICCs for total nightly ODBA were highest (implying greater night-to-night consistency) in autumn (0.57) but also relatively high in spring and summer (both 0.35); for total nightly activity duration, spring and autumn ICCs were high (0.37 and 0.38, respectively) and somewhat lower in summer (0.27). These ICCs indicate substantial differences between individual activity regimes not explicable by the suite of modeled drivers of hourly ODBA, with a 1.25-, 1.49-, and 1.92-fold difference in average nightly ODBA expenditure from the most to the least ODBA-intensive badger for spring, summer, and autumn, respectively (Figure 7a and Figure 8), with the most active badgers in spring, summer, and autumn being active for 2.2, 1.9, and 3.2 h a night, respectively, longer than the least (Figure 7b). For individuals with data for multiple seasons, there was no intra-individual correlation between spring and summer individual intercepts (Spearman rank correlation = 0.07 and −0.02 for total ODBA and minutes spent active, respectively); that is, intra-individual consistency of mechanical energy expenditure tactics did not extend between seasons (sample size was insufficient to evaluate correlations with autumn intercepts).

### 3.5. Diet

**Figure 9:**
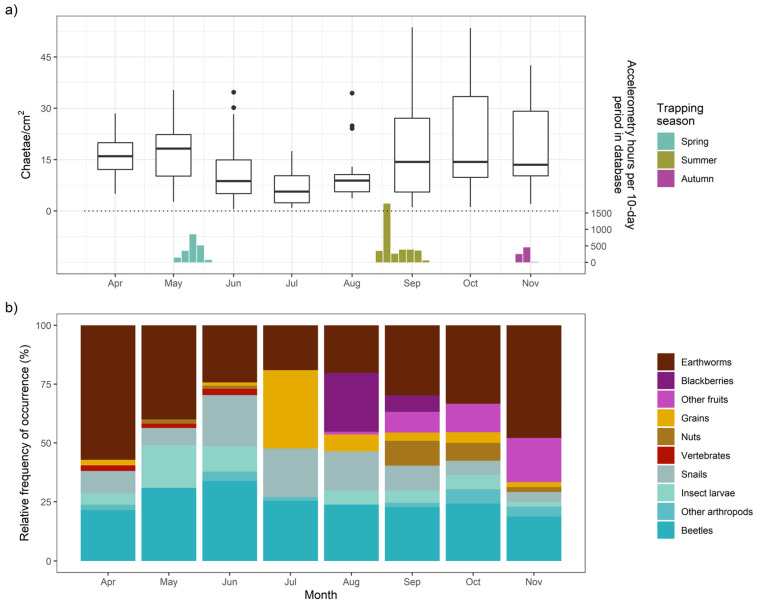
Seasonal diet trends. (**a**) Average chaetae count in 10 1-cm 2 squares for each fecal sample collected and analyzed, with bar plots indicating the timing and relative frequency of corresponding seasonal accelerometry data. Two observations from October were excluded from the plot due to particularly high chaetae counts. (**b**) Relative frequency of occurrence of diet categories in feces from a month.

Badger diet varied opportunistically throughout 2018 (Figure 9). Earthworms were the primary prey item consumed by this population, with 77.9% average monthly FO (and 96% FO in March)—alongside year-round consumption of arthropods and snails (Figure 9b)—but earthworm consumption was substantially lower during the summer (Jun-Aug, Figure 9a), with cultivated wheat (*Triticum aestivum var.*, FO = 76% in Jul) and wild blackberries (*Rubus fruticosus*, FO = 84% in Aug) filling some of this deficit. By September, blackberries were supplemented with other fruits (FO = 32% in Sep), hazelnuts (*Corylus avellana*), horse chestnuts (*Aesculus hippocastaneum*), and sweet chestnuts (*Castanea sativa*), which continued to be substantial diet components through the resumption of high earthworm consumption in autumn (Figure 9b).

## 4. Discussion

We observed substantial inter-individual heterogeneity in daily ODBA and activity expenditure on the same day at the same site, and therefore under the same environmental and social conditions, with some individuals expending almost twice as much mechanical energy as others (Figure 1). Moreover, per individual, these different energy expenditure tactics remained consistent within a season. Drivers explaining ODBA expenditure were complex (Figure 3), involving responses at the hourly level through to season-specific weather effects (Figure 4), effects of life-history (Figure 3 and Figure 5), and differential plasticity to prevailing weather according to individual body condition (Figure 6).

Badgers predominantly consumed earthworms in spring (Figure 9), which surface in an optimal mild temperature and humidity range [57]. Accordingly, in spring, activity increased linearly with hourly temperature, while ODBA peaked at intermediate temperatures (optimum determined by BCI, see Figure 6) and at precipitation levels of 0.8 mm/h. Being active during cooler and wetter conditions, exacerbated by wind chill (decreasing ODBA by up to 24.2% under the windiest conditions) [20], would be expected to incur higher thermoregulatory costs [48,75]. Strong winds may also disrupt badgers’ ability to use scent or hearing to detect earthworms [57]. These threshold-bounded effects of spring temperature and precipitation mirror non-linear relationships detrimental to badger body condition and survival [30,56], with high precipitation likely causing heat loss due to fur-soaking [76]. While shallow soil temperature (SoilT_10_) in spring was not related to either ODBA or activity (despite including a spring with unusually warm SoilT_10_), warmer SoilT_30_ was associated with up to a 23.1% decrease in ODBA.

During the summer deployments, ODBA was again affected positively by temperature up to an optimum before declining (again, optimum determined by BCI). Unlike in spring, summer activity exhibited the same tipping point effect as ODBA, decreasing beyond 13.1 °C (only slightly higher than the summer long-term average temperature, Table S1). We observed no effect of rainfall in summer; however, in contrast to spring, there was up to a 19.4% increase in activity probability under the windiest conditions, but with no effect on ODBA. In summer, ODBA decreased by up to 28.1% with warmer SoilT_10_ conditions, which also reduced activity; in contrast, the highest SoilM conditions were associated with a 1.4-fold ODBA increase. We propose that moister, milder soil conditions, likely promoting earthworm availability, drove significant facultative ODBA increases; however, badgers depend less on earthworms in their summer diet and instead consume a greater proportion of berries and cereal crops (Figure 9)—for which availability is not affected by weather conditions. Unnecessary exertion in high ambient temperatures can lead to overheating, a major stressor for wild animals [77]. Therefore, heat often causes a hyperthermic lethargy response [78,79] in order to avoid morbidity [80]. These various summer weather effects suggest that, when freed from weather driving the availability of their primary food, badgers primarily sought instead to stay cool, reducing both ODBA and activity when both the air and soil were warmer, and benefitting from a cooling breeze. Heat stress may be particularly pertinent to badgers, which evolved primarily as a cold-climate species [81] and exhibit various physiological adaptations to minimize thermal losses [52].

In autumn, ODBA and activity were less responsive to weather, especially air temperature, than in other seasons—although likely caused in part by the smaller autumnal sample size. Of the soil covariates tested, only SoilT_10_ (but not SoilT_30_; and the effects of SoilM fell below significance after accounting for autocorrelation) had a significant effect on activity metrics. This influence was, however, substantial, with a 2.3-fold increase in ODBA and a 44% increase in activity when soils were warmest—likely linked to conditions under which earthworms surfaced in the autumn (especially as SoilT_10_ was significantly cooler during the study period, *p* < 0.001, than the long-term mean for the same dates, Table S1). Badgers also avoided heavy rain in the autumn, which reduced activity by up to 25.6%, corroborating patterns reported by Noonan et al. [49]. Henry [82] reported that with earthworm scarcity on frosty nights, the effectiveness of badger foraging was over ten times lower than during warmer nights, and thus badgers must achieve a minimum autumnal body by using fat reserves to support torpor. Accordingly, only badgers in low body condition—those motivated to go foraging even under the low likelihood of reward—tend to remain relatively active during years with poor autumn foraging conditions [49].

Individual badgers exhibit heterogeneous investments in social activity throughout the year [43], with some individuals engaging in more energy-intensive life-history strategies than others [25,55,83]. Within the overarching context of extrinsic effects, we detected no effect of sex on ODBA, although the limited number of contrasts in our dataset precluded rigorous analysis on the base of sex. However, we did find different relationships between BCI and springtime activity between sexes. Higher-condition females were less likely to be active than lower-condition ones in the spring. While the highest-BCI female badger in the dataset (the only one not to lactate that year) contributed an influential data point for this relationship, the interaction term remained significant after excluding it.

Reproduction takes a well-documented toll on female badger condition [84], and unless females gain back lost body condition they suffer reduced survival and reproduction probability during the next winter [25]. Therefore, the higher activity seen in low-BCI reproductive females implies that the residual effects of reproductive investments reduce an individual’s energy budget flexibility. This elevated maternal activity rate may relate to the slow rate of social integration for badger cubs relative to other social carnivores [85], during which time they are vulnerable to infanticide [86], requiring mothers to forego foraging to safeguard their cubs unless in particularly poor condition [59,87]. In contrast, males showed a (slight) positive BCI–activity relationship (Figure 5), possibly reflecting that, in the absence of paternal care, males in good condition could spend more time engaging in “optional” social behavior such as visiting other setts. Note, however, that welfare considerations (a legal closed season, linked to not depriving neonatal cubs of maternal care) prohibited us from trapping and collaring badgers during the peak post-partum mating period in mid-February [88]—when sex-based activity might differ most [50].

ODBA was clearly related to substantial inter-individual differences in plasticity tied to body condition: in spring, higher-BCI badgers expended more ODBA with warmer temperatures while lower-BCI badgers exhibited consistently higher ODBA irrespective of temperature (Figure 6a). Higher BCI appeared to provide individuals with some degree of buffering, enabling them to undertake less energy expenditure on cool spring nights, whereas lower-BCI individuals foraged even under sub-optimal temperature conditions (Figure 4). Individuals in poor condition need to prioritize foraging activity irrespective of poor foraging conditions [12], particularly during energy-intensive life-history stages (e.g., lactation, breeding) [16,89], possibly at a cost to social investments such as inter-sett visits [43,90].

The BCI-plasticity relationship reversed during summer (Figure 6b). Badgers with higher BCIs decreased ODBA beyond an inflection point, implying that higher-BCI individuals may seek to avoid excess activity when well-insulated with fat, given that hyperthermia risks pathophysiological effects [91]. Not least, insensible water loss due to panting to thermoregulate [92,93] is harder to replace in summer when puddles of water are scarce; access to water is a major constraint on badgers in arid conditions such as are found around the Mediterranean [94]. While diurnal animals can partially compensate for warming conditions by shifting activity towards nocturnality [95], nocturnal ones such as badgers cannot adapt regimes further and must cope by reducing mechanical expenditure [96].

Age effects also suggested that warm summer conditions may constrain mechanical energy expenditure in badgers. Older badgers were less likely to be active than young badgers and expended less ODBA during summer nights. Heat stress generally has more severe effects on elderly individuals, including humans [97]. To mitigate this stress, hyperthermia triggers lethargy, largely through the generation of central fatigue involving changes in dopamine and serotonin levels [79], reducing activity and minimizing thermogenesis [98]. Older badgers typically carry smaller fat stores despite somatic reserves in summer elevating annual survival probability with advancing age [25]. Therefore, the observed additional activity reductions seen in older badgers likely relate to a necessary avoidance of unsustainable loss of somatic condition during the summer.

Our observation of badger activity commencing before dusk and extending beyond dawn in our May deployment corroborates previous studies [99], where badgers’ relatively fixed and incompressible nightly routines (e.g., foraging, mutual and self-grooming, scent marking, and territorial defense) [59] require longer than short nights permit. Furthermore, our fine-scale individual data revealed that from 271 badger days of activity, only 126 (46.5%) involved one nonstop bout of activity—on 123 nights (45.4%) the badger became inactive for at least one hour and then resumed activity (Figure 2); one badger even undertook four cycles of activity/inactivity in one night. While our data do not allow us to firmly discern between inactive waking and temporary polyphasic sleep (naps), the latter has been hypothesized to be facilitated by environmental conditions [100], providing an axis for future investigation. The proportion of unbroken activity was higher in spring (64.3%), during the shortest nights than in summer (35.7%) or autumn (53.3%) deployments, demonstrating that individuals tailor their energy budgeting not only from night to night but also within a night, according to environmental constraints and seasonal priorities.

Beyond the explanatory power of these intrinsic and extrinsic drivers, high intra-class correlation coefficients exposed substantial individual consistency in the duration of nightly activity and ODBA (Figure 7 and Figure 8). This suggests that energy budgeting typologies may exist that persist at least within seasons, implying that once committed to an energetic tactic, badgers continued to follow it while similar seasonal conditions prevailed; switching tactics may presumably nullify or destabilize preceding energy investments. Crucially, however, even after accounting for variation attributable to sex, age, and body condition, we also found strong evidence for substantial inter-individual heterogeneity in mechanical energy investment tactics across the population. That is, given the exact same overarching conditions, two similar individuals might exhibit substantially different ODBA/activity profiles (Figure 8).

In addition to mechanical energy expenditure, an individual’s total energy budget is also comprised by obligate metabolic processes not measured by accelerometry [13], including homeostasis [101], immune responses [102], and thermoregulation [103]. Superficially similar individuals may therefore have substantially different underlying metabolic rates or sustain different metabolic costs, which may factor into their activity tactics [104]. Moreover, inter-individual differences in the security with which basal energy inputs are met could lead to heterogeneous investment in “surplus” activities, such as exploration [105], social interactions [106], territorial defense [107], sexual advertisement and other forms of communication [108], *inter alia*. For instance, high-BCI badgers in the studied population perform more inter-sett visits than do low-BCI badgers [43], implying that badgers experiencing higher energy constraints reduce non-essential activities. Importantly too, many animals utilize inactivity (both sleep and torpor) [109,110] to reduce energy expenditure [111]. This is particularly important because—at least in humans [112]—it is the combination of low energy intake and low energy expenditure (low energy flux), not energy surplus, that predicts future body fat gain.

Niche variation [113] between superficially similar individuals may also contribute to tactical heterogeneity. We selected badgers from relatively large social groups (avg. of 10.1 badgers from 2011 to 2016, vs. avg of 4.3 in other social groups), even within the context of this high-density population [46], where higher density militates for trophic niche specialization [114]. As the diets of individuals become more uniquely specialized, the cumulative niche breadth of a population increases [7,115], suggesting a greater capacity for inter-individual energetic heterogeneity.

Although plasticity to environmental change is generally associated with population resilience [24,116], because individuals are simultaneously responding differently to existential selection pressures, it comes with costs to efficiency [117,118]. Hypothetically, in stable environments, a single optimal strategy should become canalized; but for a generalist omnivore with a variable social system such as the badger [59], and in a seasonally temperate mosaic habitat (a multidimensional niche) [119], a single optimal solution to maximal fitness is not achievable—as opposed to for species with a more specialized diet, or that benefit from group hunting, which promotes higher activity fidelity among conspecifics [120]. Within populations with a wide niche breadth, substantial energy-balancing diversity can influence pace-of-life syndromes (POLS) [121] through the co-variation in behavioral and physiological traits [122,123]. In this studied badger population, there is evidence of demographic [83], developmental [124], and endocrinological [55] variation in pace-of-life. While here we cannot link to POLS directly, these syndromes may also contribute to inter-individual differences in how badgers expend energy under the same conditions. For diverse energetic tactics to persist within a population, each must either provide equivalent fitness or those that underperform under one environmental scenario must have a selective fitness advantage under different conditions [125]. As energy availability can vary substantially as a function of environmental stochasticity, individuals should seek to minimize their exposure to fitness variance, through “bet-hedging” [23,126]. Consequently, patterns emerge when fluctuating selection pressures [127] alternatively favor suites of energy expenditure tactics that cluster individuals into high- and low-competition phenotypes [128].

## 5. Conclusions

Rapid environmental change—particularly climate change—exerts a disruptive selection pressure on populations [129], likely impacting which energy expenditure tactics succeed or fail under novel conditions [24]. Badgers, as an emblematic mesocarnivore with a wide bioclimatic niche [130], provide fertile ground for examining the latent capacity of species to adopt in situ behavioral coping tactics to conform to these anthropogenic selective stressors. In this analysis, we unveiled a delicate patchwork of individual heterogeneity in energetic response to environmental triggers, dependent on reproduction, age, and body condition. Ultimately, the persistence of many non-vagile populations in the Anthropocene will depend on whether parallel patchworks of behavioral responses are a match for novel energetic challenges. Our findings would compel the wise conservation practitioner to consider not only emerging environmental changes, but also how these interact with different life history stages and the diverse phenotypes within wild populations, so that they may better anticipate critical points in population and species viability.

## Figures and Tables

**Table 1:** Deployments of tri-axial accelerometry collars.

Season	Date Deployed	Deployment Duration	# Deployed	# Recovered	# Returning Data	Avg. Data Returned
Spring	21/05/18	7 days	9	7	5	7.02 days
Summer	03/09/18	7–10 days	10	10	10	6.62 days
Summer	09–10/09/18	65–82 days	9	6	4	15.50 days
Autumn	13/11/18	6–8 days	12	11	0	0 days
Spring	27/05/19	7–8 days	12	12	12	7.04 days
Summer	02/09/19	7–9 days	12	12	10	7.98 days
Autumn	13/11/19	6–8 days	11	11	10	4.51 days

## Data Availability

The datasets used in this study will be stripped of unique identifying information and made available at the Wytham Woods Badger Project dataverse (https://dataverse.harvard.edu/dataverse/wytham-badgers) accessed 13 April 2025 upon acceptance for publication.

## Supplementary Materials

The following supporting information can be downloaded at https://www.mdpi.com/article/10.3390/ani15111560/s1. Supplementary Materials contains: S1. an evaluation of weather covariates within seasonal deployments; S2. deviations from long-term average and within-season collinearity; S3. methods for determining an accelerometry threshold for activity; S4. model selection comparing approaches to handling autocorrelation diet analysis methods [49,64,65,69,73,131,132,133,134,135,136,137,138]. Supplementary Tables: Table S1: Weather covariate characterizations; Table S2: Top model coefficients for seasonal ODBA model selection approaches; Table S3.1. Models with ΔAIC < 2 from top model for all-subsets selection with AR-1 term for hourly springtime ODBA; Table S3.2. Models with ΔAIC < 2 from top model for all-subsets selection with no accounting for autocorrelation for hourly springtime ODBA; Table S3.3. Models with ΔAIC < 2 from top model for all-subsets selection on halved data for hourly springtime ODBA; Table S3.4. Models with ΔAIC < 2 from top model for all-subsets selection with AR-1 term for hourly summertime ODBA; Table S3.5. Models with ΔAIC < 2 from top model for all-subsets selection with no accounting for autocorrelation for hourly summertime ODBA; Table S3.6. Models with ΔAIC < 2 from top model for all-subsets selection on halved data for hourly summertime ODBA; Table S3.7. Models with ΔAIC < 2 from top model for all-subsets selection with AR-1 term for hourly autumn ODBA; Table S3.8. Models with ΔAIC < 2 from top model for all-subsets selection with no accounting for autocorrelation for hourly autumn ODBA; Table S3.9. Models with ΔAIC < 2 from top model for all-subsets selection on halved data for hourly autumn ODBA; Table S3.10. Models with ΔAIC < 2 from top model for all-subsets selection for hourly springtime activity; Table S3.11. Models with ΔAIC < 2 from top model for all-subsets selection for hourly summertime activity; Table S3.12. Models with ΔAIC < 2 from top model for all-subsets selection for hourly autumn activity. Supplementary Figures: Figure S1.1: Correlations between covariates during the spring study period; Figure S1.2: Correlations between covariates during the summer study period; Figure S1.3: Correlations between covariates during the autumn study period; Figure S2: Distribution of active/inactive samples.
