# Supplementary material for: Individual Mechanical Energy Expenditure Regimens Vary Seasonally with Weather, Sex, Age and Body Condition in a Generalist Carnivore Population: Support for Inter-Individual Tactical Diversity
Source: Animals (Basel). 2025 May 27;15(11):1560. doi: 10.3390/ani15111560 (PMC12153667; doi:10.3390/ani15111560)
Supplement: Supplementary file 1 [file animals-15-01560-s001.zip › animals-3616451-supplementary.pdf]

Supplementary Material

Individual Mechanical Energy Expenditure Regimens Vary Seasonally with Weather, Sex, Age and Body Condition in a Generalist Carnivore Population: Support for Inter-individual Tactical Diversity

Julius G. Bright Ross, Andrew Markham, Michael J. Noonan, Christina D. Buesching, Erin Connolly, Denise W. Pallett, Yadvinder Malhi, David W. Macdonald*, Chris Newman

*** Correspondence:** David. W. Macdonald: david.macdonald@biology.ox.ac.uk

1.1 Evaluation of weather covariates within seasonal deployments: Deviations from long-term average and within-season collinearity

Firstly, we evaluated whether the weather covariates we tested differed significantly or substantially from the long-term mean (1991-2015, from Rennie et al., 2017) for this study site. To do so, we performed seasonal one-way ANOVA differences-of-means tests (2018 or 2019 vs. 1991-2015) for hourly weather metrics between sunset and sunrise (*suncalc* package: Benoit and Achraf, 2019) for the dates with recorded accelerometry data. In the case of hourly air temperature (Temp), we controlled for the contribution of the day-night cycle by using a generalized additive model (GAM, *mgcv* package: Wood and Scheipl, 2020) with a 5-k spline for hour (1-24). The long-term mean, annual deviations from this mean (from models), and long-term standard deviation are provided for each covariate in Table S1.1.

For each seasonal hourly ODBA model selection, we carried out an independent assessment of collinearity between explanatory variables. To do so, we used correlations between standardized predictors (mean = 0, SD = 1), and considered any covariates with R values of 0.6 or greater to be too collinear to include in the same model. Figures S1.1-S1.3 were constructed using the *corrplot* package in R (Wei and Simko, 2017).

1.2 Determining an accelerometry threshold for activity

Overall dynamic body acceleration (ODBA) is a multi-modally distributed metric, with most animal time budgets breaking down into two primary "modes": those times when individuals are active, and when they are not. The inactive mode lies near 0; the active mode lies at some positive non-zero value that will be determined by study-specific parameters (individual conditions, extrinsic conditions, species, etc.). Non-zero values can still be confidently assigned to "inactivity" if they represent sampling noise or low-level motion from respiration, especially at a fine timescale (Noonan et al., 2014). Some threshold value T separates the two distributions, where activity in minute k (a_k) is equal to the signum function (1 for positive values; 0 for negative ones) of the difference between that minute's ODBA score and T:

$$a_k = sgn(ODBA_k - T)$$

We determined the value of *T* through sensitivity analysis: we found the value of *T* where the above equation exhibited the lowest sensitivity (smallest difference in the proportion of minutes classified as "active") to a change (± 0.05) in the value of *T*. For our study, across all badgers in all seasons, this produced a value of *T* equal to 0.278, which partitioned our data into 63.4% "inactive" and 36.6% "active" minutes (Fig. S2.1, n = 390,300).

1.3 Model selection comparing approaches to handling autocorrelation

We compared three separate approaches to selecting a top model for seasonal hourly ODBA. The first of these was to perform all-subsets selection on models incorporating an AR-1 autoregressive structure (*gamm4* library: Wood and Scheipl, 2020). The second approach was to perform all-subsets selection without accounting for autocorrelation. The third was to thin these data and perform all-subsets selection on these thinned data. To thin these data, we created a tracker that began each badger's data on a different number from 1-10. We then continued the sequence to 10, then starting again at 1, until that badger's hourly ODBA data had been numbered start-to-finish. Then, we could create 1/2, 1/5, and 1/10-scale datasets without concerns relating to over-sampling a specific time of day for any given badger, and without under-sampling any hours in the dataset. After examining autocorrelation function (ACF) estimates of top models, all relevant autocorrelation was accounted for after halving these data, so we did not proceed with all-subsets selection of 1/5 and 1/10-scale datasets.

As can be seen in Table S3.1, all-subsets selection on the original dataset not only inflated term significance, but also led to the selection of additional terms that no longer retained significance once autocorrelation had been accounted for. Halving these data produced comparable selection of terms to the other two approaches but showed a tendency to produce terms with less fidelity to the other estimates and consistently retained marginally significant or non-significant terms. Therefore, we proceeded in the main text analyses by reporting the results from all-subsets selection on models with AR-1 terms, which produced similar estimates to those of models without an autoregressive term, although it but proved more conservative in its selection of terms.

1.4 Diet analysis methods

Forty-one latrines within ~100 meters of five social-group sett clusters (see Buesching et al., 2016 for background on social-group sett clusters) were examined once a month from April to November 2018, from which fecal samples were collected. On the first day of each sampling period, the freshest feces found at each latrine were collected, and a light layer of sand was sprinkled over the remaining feces (in order to ensure that, subsequently, only the freshest samples were collected), taking care to switch gloves between latrines (after Kilshaw et al., 2009). Latrines were then visited for three days and up to two fresh feces collected per sett per day, again sprinkling any surplus samples. Five samples were analyzed for each social-group sett cluster per month for dietary content (n = 25/month, total = 200). Each sample was weighed, oven-dried for 48 hours, and weighed again, then dissolved in approx. 1L of water, before being rinsed through a 0.5 mm stainless steel sieve, large enough to allow earthworm chaetae to flow through (Cleary et al., 2009; Kaneko et al., 2006). Earthworm chaetae in badger feces correlate with the number of earthworms consumed (Kruuk and Parish, 1981) and have therefore been used in various studies to quantify the role of earthworms in badger diets (e.g., Balestrieri et al., 2009; Goszczyński et al., 2000; Kaneko et al., 2006; Zabala and Zuberogoitia, 2003). After Kruuk and Parish (1981), the rinsing effluent was allowed to settle for ten minutes and 1.5 mm of sediment was drawn up from the bottom and examined in a petri dish; ten 1-cm^2 squares

were then examined under 10x magnification and the number of chaetae in each were averaged. The sieved material was examined for other diet items, and these were sorted and once again oven-dried. Dry weights were recorded for each diet item, as well as for the remaining matrix. Fragments of beetle elytra were counted and summed, rounding up to whole beetles where applicable. In some cases, diet items comprised a significant enough fraction of the matrix that a subsample was taken, the diet item in question completely sorted from it, and weighed. The fraction of the subsample matrix the diet item comprised was considered to be representative of the entire matrix.

Occurrence was defined differently for different diet items. For earthworms, a score of "2" or higher on the scale provided in Kruuk and Parish (1981) was used as a minimum requirement for occurrence, representing at least 6 chaetae/cm^2 in a sample's wash-through. For beetles, detection of at least one eighth of an elytron or one leg was sufficient to register occurrence; for other arthropods, occurrence of some digested arthropod material that was identifiably not coleopteran qualified for occurrence. For all other categories, a non-zero weight of the given category was taken to indicate occurrence.

Frequency of occurrence (FO, occurrences/total samples) and relative frequency of occurrence (RFO, occurrences/total occurrences of all diet items) were calculated monthly for each diet item. We then summed RFO for related diet objects (e.g., hymenoptera + dermaptera = "non-beetle arthropods").

References

Balestrieri, A., Remonti, L., Prigioni, C., 2009. Exploitation of food resources by the Eurasian badger (*Meles meles*) at the altitudinal limit of its Alpine range (NW Italy). Zoolog. Sci. 26, 821–827. https://doi.org/10.2108/zsj.26.821

Benoit, T., Achraf, E., 2019. suncalc: Compute sun position, sunlight phases, moon position and lunar phase. R package version 0.5.0.

Buesching, C.D., Newman, C., Service, K., Macdonald, D.W., Riordan, P., 2016. Latrine marking patterns of badgers (*Meles meles*) with respect to population density and range size. Ecosphere 7, e01328.

Cleary, G.P., Corner, L.A.L., O'Keeffe, J., Marples, N.M., 2009. The diet of the badger *Meles meles* in the Republic of Ireland. Mamm. Biol. 74, 438–447. https://doi.org/10.1016/j.mambio.2009.07.003

Goszczyński, J., Jędrzejewska, B., Jędrzejewski, W., 2000. Diet composition of badgers (*Meles meles*) in a pristine forest and rural habitats of Poland compared to other European populations. J. Zool. 250, 495–505. https://doi.org/10.1111/j.1469-7998.2000.tb00792.x

Kaneko, Y., Maruyama, N., Macdonald, D.W., 2006. Food habits and habitat selection of suburban badgers (*Meles meles*) in Japan. J. Zool. 270, 78–89. https://doi.org/10.1016/j.jtbi.2005.08.029

Kilshaw, K., Newman, C., Buesching, C., Bunyan, J., Macdonald, D.W., 2009. Coordinated latrine use by European badgers, *Meles meles*: Potential consequences for territory defense. J. Mammal. 90, 1188–1198. https://doi.org/10.2307/27755113

Kruuk, H., Parish, T., 1981. Feeding specialization of the European badger *Meles meles* in Scotland.

J. Anim. Ecol. 50, 773–788.

Noonan, M.J., Markham, A., Newman, C., Trigoni, N., Buesching, C.D., Ellwood, S.A., Macdonald, D.W., 2014. Climate and the individual: Inter-annual variation in the autumnal activity of the European badger (*Meles meles*). PLoS One 9, e83156. https://doi.org/10.1371/journal.pone.0083156

Rennie, S.., Adamson, J., Anderson, R., Andrews, C., Bater, J., Bayfield, N., Beaton, K., Beaumont, D., Benham, S., Bowmaker, V., Britt, C., Brooker, R., Brooks, D., Brunt, J., Common, G., Cooper, R., Corbett, S., Critchley, N., Dennis, P., Dick, J., Dodd, B., Dodd, N., Donovan, N., Easter, J., Eaton, E., Flexen, M., Gardiner, A., Hamilton, D., Hargreaves, P., Hatton-Ellis, M., Howe, M., Kahl, J., Lane, M., Langan, S., Lloyd, D., McCarney, B., McElarney, Y., McKenna, C., McMillan, S., Milne, F., Milne, L., Morecroft, M., Murphy, M., Nelson, A., Nicholson, H., Pallett, D., Parry, D., Pearce, I., Pozsgai, G., Rose, R., Schafer, S., Scott, T., Sherrin, L., Shortall, C., Smith, R., Smith, P., Tait, R., Taylor, C., Taylor, M., Thurlow, M., Turner, A., Tyson, K., Watson, H., Whittaker, M., Wilkinson, M., Wood, C., 2017. UK Environmental Change Network (ECN) meteorology data: 1991-2015. https://doi.org/10.5285/fc9bcd1c-e3fc-4c5a-b569-2fe62d40f2f5

Wei, T., Simko, V., 2017. R package “corrplot”: Visualization of a correlation matrix.

Wood, S., Scheipl, F., 2020. gamm4: Generalized Additive Mixed Models using “mgcv” and “lme4.”

Zabala, J., Zuberogoitia, I., 2003. Badger, *Meles meles* (Mustelidae, Carnivora), diet assessed through scat-analysis: A comparison and critique of different methods. Folia Zool. 52, 23–30.

1 Supplementary Tables

Table S1 **Weather covariate characterizations.** Deviation shown in same units as average and SD. Significance levels shown using standard asterisk notation: ***$p < 0.001$; **$0.001 \leq p < 0.01$; *$0.01 \leq p < 0.05$. Bold script cells indicate when seasonal deviation for a covariate equals or exceeds long-term standard deviation.

		**Spring**			**Summer**			**Autumn**		
	Year	Average 1991-2015	Deviation	SD 1991-2015	Average 1991-2015	Deviation	SD 1991-2015	Average 1991-2015	Deviation	SD 1991-2015
Temp.	2018	10.1 °C	+2.3***	2.9 °C	12.1 °C	+0.1	2.7 °C	-	-	-
	2019	11.0 °C	+0.1	2.7 °C	12.9 °C	-1.4***	2.6 °C	6.1 °C	-1.9***	3.6 °C
Rainfall	2018	0.10 mm	+0.15*	0.47 mm	0.08 mm	-0.02	0.48 mm	-	-	-
	2019	0.08 mm	-0.05	0.38 mm	-	-	-	0.07 mm	+0.09*	0.34 mm
Wind speed	2018	1.84 m/s	+0.13	0.97 m/s	1.66 m/s	+0.23***	0.92 m/s	-	-	-
	2019	1.64 m/s	+0.02	0.86 m/s	1.60 m/s	-0.16	0.87 m/s	2.22 m/s	-0.29*	1.34 m/s
Soil temp. (10 cm)	2018	**14.5** °C	**+2.3*****	**2.3** °C	15.5 °C	-0.1	1.8 °C	-	-	-
	2019	15.7 °C	-0.5*	2.1 °C	16.7 °C	-1.4***	1.6 °C	7.5 °C	-1.6***	2.0 °C
Soil temp. (30 cm)	2018	14.1 °C	+0.6**	1.3 °C	16.0 °C	-0.2*	1.2 °C	-	-	-
	2019	15.1 °C	-0.7***	1.3 °C	16.8 °C	-0.9***	1.0 °C	8.8 °C	-0.9***	1.5 °C
Soil moisture	2018	31.1%	-2.8**	7.3%	25.7%	-4.0***	6.8%	-	-	-
	2019	**30.1%**	**-10.8*****	**8.2%**	25.8%	-5.2%***	7.2%	35.4%	+3.2%***	5.2%
Relative humidity	2018	84.8%	+6.7***	10.2%	88.0%	+0.1%	8.9%	-	-	-
	2019	86.8%	+2.3	10.3%	87.1%	-1.4%	9.6%	92.1%	+2.7%***	7.0%

Table S2: **Top model coefficients for seasonal ODBA model selection approaches.** All covariates are standardized within-season to mean = 0, standard dev. = 1. Terms not included in top model indicated with a dash ("-"); terms not evaluated in model selection indicated with "NA". A spline for hour was significant in all models and is not shown here. "Sex" represents a binomial factor, where the baseline sex is male. Significance levels shown using standard asterisk notation: ***$p < 0.001$; **$0.001 \leq p < 0.01$; *$0.01 \leq p < 0.05$; **.**$0.05 < p < 0.10$.

	Spring			Summer			Autumn		
	**AR-1**	Null	Halved	**AR-1**	Null	Halved	**AR-1**	Null	Halved
Intercept	**0.003**	-0.043	-0.026	**-0.002**	-0.009	0.001	**-0.006**	-0.007	-0.02
Temp.	**0.046***	0.047***	0.025	**0.067****	0.06***	0.097 ***	-	0.083**	0.097*
Temp.^{2}	**-0.027***	-0.031**	-0.035 *	**-0.033***	-0.034***	-0.032*	-	-0.099***	-0.085*
Rainfall	**-0.024***	-0.027*	-0.029 .	-	-	0.019	-	-	-
Rainfall^2	**-0.018.**	-0.022*	-	-	-	-	-	-	-
Wind speed	**-0.042****	-0.039***	-0.027.	-	0.018	-	-	-	-
Relative humidity	-	-	-	-	-	-	-	-	-
Soil temp. (10 cm)	-	-	-	-	-	-	**0.099***	-	-
Soil temp. (30 cm)	**-0.047****	-0.046***	-0.047**	**-0.043****	-0.043***	-0.042 **	-	0.043.	0.053
Soil moisture	-	0.034*	0.037*	**0.042****	0.041***	0.045 **	-	-	-
Age	-	-	-	**-0.07*****	-0.072***	-0.075***	-	-0.102	-0.091.
BCI	**-0.054***	0.029	0.057	**0.012**	-0.002	0.006	-	-0.044	-
Sex	-	0.078*	0.105**	-	-	-	NA	NA	NA
BCI: Temp.	**0.031***	0.032***	0.035**	**-0.042****	-0.046***	-0.051***	-	-0.049.	-
BCI: Rainfall	-	0.028.	0.037	-	-	-	-	-	-
BCI:Sex	-	-0.075*	-0.070.	-	-	-	NA	NA	NA

Table S3.1. **Models with ΔAIC < 2 from top model for all-subsets selection with AR-1 term for hourly springtime ODBA.** All covariates are standardised within-season to mean = 0, standard dev. = 1. A spline for hour was significant in all models, and is not shown here, nor is intercept. " Sex" represents a binomial factor, where the baseline sex is male.

Temp.	$Temp.^2$	Rainfall	$Rainfall^2$	Wind speed	Relative humidity	Soil temp. (10 cm)	Soil temp. (30 cm)	Soil moisture	Age	BCI	Sex	BCI:Temp.	BCI:Rainfall	BCI:Sex	ΔAIC
0.046	-0.027	-0.024	-0.018	-0.042	-	-	-0.047	-	-	-0.054	-	0.031	-	-	0
0.044	-0.026	-0.024	-0.017	-0.038	-	-	-0.047	0.033	-	0.021	0.076	0.03	-	-0.072	0.31
0.046	-0.028	-0.023	-0.018	-0.042	-	-	-0.046	-	-	-0.019	0.046	0.03	-	-	0.53
0.041	-0.028	-0.025	-	-0.041	-	-	-0.047	-	-	-0.054	-	0.03	-	-	1.18
0.039	-0.026	-0.025	-	-0.037	-	-	-0.047	0.034	-	0.022	0.076	0.03	-	-0.073	1.27
0.046	-0.027	-0.026	-0.018	-0.041	-	-	-0.047	-	-	-0.052	-	0.032	0.014	-	1.35
0.044	-0.028	-0.022	-0.018	-0.043	-	-	-0.044	-	-	-0.005	0.052	0.03	-	-0.041	1.35
0.046	-0.026	-0.024	-0.018	-0.039	-	-	-0.048	0.018	-	-0.011	0.056	0.03	-	-	1.44
0.043	-0.026	-0.026	-0.017	-0.038	-	-	-0.047	0.034	-	0.024	0.078	0.032	0.016	-0.073	1.47
0.047	-0.027	-0.024	-0.018	-0.042	-	-	-0.047	-	0.016	-0.047	-	0.031	-	-	1.53
0.047	-0.026	-0.024	-0.018	-0.04	-	-	-0.048	0.012	-	-0.054	-	0.031	-	-	1.55
0.04	-0.028	-0.025	-	-0.041	-	-	-0.046	-	-	-0.019	0.046	0.03	-	-	1.73
0.045	-0.027	-0.025	-0.018	-0.042	-	-	-0.046	-	-	-0.016	0.047	0.032	0.015	-	1.81
0.046	-0.028	-0.023	-0.018	-0.042	-	-	-0.046	-	0.019	-0.009	0.048	0.031	-	-	1.84
0.044	-	-0.025	-0.017	-0.036	-	-	-0.05	0.036	-	0.021	0.076	0.031	-	-0.073	1.9

Table S3.2. **Models with ΔAIC < 2 from top model for all-subsets selection with no accounting for autocorrelation for hourly springtime ODBA.** All covariates are standardised within-season to mean = 0, standard dev. = 1. A spline for hour was significant in all models, and is not shown here, nor is intercept. " Sex" represents a binomial factor, where the baseline sex is male.

Temp.	Temp.^{2}	Rainfall	Rainfall^2	Wind speed	Relative humidity	Soil temp. (10 cm)	Soil temp. (30 cm)	Soil moisture	Age	BCI	Sex	BCI:Temp.	BCI:Rainfall	BCI:Sex	ΔAIC
0.047	-0.031	-0.027	-0.022	-0.039	-	-	-0.046	0.034	-	0.029	0.078	0.032	0.028	-0.075	0
0.048	-0.031	-0.022	-0.023	-0.04	-	-	-0.046	0.032	-	0.021	0.073	0.029	-	-0.072	1.43
0.049	-0.032	-0.025	-0.024	-0.043	-	-	-0.045	-	-	-0.008	0.052	0.032	0.027	-	1.64
0.05	-0.032	-0.026	-0.024	-0.043	-	-	-0.046	-	-	-0.045	-	0.032	0.026	-	1.67
0.048	-0.031	-0.027	-0.023	-0.04	-	-	-0.047	0.019	-	0.004	0.066	0.032	0.028	-	1.93
0.047	-0.031	-0.027	-0.022	-0.039	-	-	-0.046	0.033	0.005	0.03	0.078	0.032	0.028	-0.073	1.94

Table S3.3. **Models with ΔAIC < 2 from top model for all-subsets selection on halved data for hourly springtime ODBA.** All covariates are standardised within-season to mean = 0, standard dev. = 1. A spline for hour was significant in all models, and is not shown here, nor is intercept. “ Sex” represents a binomial factor, where the baseline sex is male.

Temp.	Temp.^{2}	Rainfall	Rainfall^2	Wind speed	Relative humidity	Soil temp. (10 cm)	Soil temp. (30 cm)	Soil moisture	Age	BCI	Sex	BCI:Temp.	BCI:Rainfall	BCI:Sex	ΔAIC
0.025	-0.035	-0.029	-	-0.027	-	-	-0.047	0.037	-	0.057	0.105	0.035	0.037	-0.07	0
0.03	-0.035	-0.02	-0.019	-0.029	-	-	-0.047	0.034	-	0.048	0.1	0.032	-	-0.068	0.47
0.025	-0.035	-0.022	-	-0.028	-	-	-0.047	0.035	-	0.05	0.102	0.032	-	-0.07	0.54
0.029	-0.034	-0.026	-0.016	-0.028	-	-	-0.047	0.035	-	0.054	0.103	0.035	0.032	-0.069	0.58
0.031	-0.037	-	-	-0.032	-	-	-0.048	0.032	-	0.05	0.102	0.032	-	-0.07	0.76
0.014	-0.032	-0.034	-	-	-	-	-0.047	0.043	-	0.061	0.108	0.035	0.038	-0.073	0.85
0.027	-0.037	-0.027	-	-0.032	-	-	-0.045	-	-	0.015	0.072	0.035	0.036	-	0.9
0.032	-0.037	-0.017	-0.021	-0.034	-	-	-0.045	-	-	0.007	0.069	0.033	-	-	0.97
0.027	-0.035	-0.03	-	-0.028	-	-	-0.049	0.023	-	0.025	0.085	0.035	0.038	-	1.15
0.031	-0.036	-0.024	-0.018	-0.033	-	-	-0.045	-	-	0.013	0.071	0.035	0.031	-	1.18
0.033	-0.039	-	-	-0.036	-	-	-0.047	-	-	0.009	0.071	0.033	-	-	1.19
0.027	-0.038	-0.02	-	-0.033	-	-	-0.045	-	-	0.008	0.07	0.032	-	-	1.37
0.014	-0.033	-0.027	-	-	-	-	-0.047	0.041	-	0.054	0.105	0.032	-	-0.073	1.53
0.032	-0.035	-0.02	-0.02	-0.03	-	-	-0.048	0.021	-	0.015	0.08	0.033	-	-	1.57
0.018	-0.032	-0.031	-0.015	-	-	-	-0.047	0.042	-	0.058	0.106	0.035	0.034	-0.072	1.6
0.031	-0.034	-0.026	-0.017	-0.029	-	-	-0.048	0.022	-	0.022	0.083	0.035	0.033	-	1.62
0.019	-0.032	-0.024	-0.019	-	-	-	-0.047	0.04	-	0.051	0.103	0.032	-	-0.072	1.65
0.027	-0.036	-0.022	-	-0.029	-	-	-0.048	0.022	-	0.017	0.081	0.032	-	-	1.79
0.031	-0.04	-	-	-0.037	-	-	-0.044	-	-	0.026	0.08	0.033	-	-0.041	1.9
0.031	-0.038	-0.016	-0.021	-0.034	-	-	-0.043	-	-	0.022	0.077	0.033	-	-0.038	1.91
0.026	-0.038	-0.026	-	-0.033	-	-	-0.043	-	-	0.029	0.079	0.035	0.036	-0.037	1.93
0.025	-0.035	-0.029	-	-0.027	-	-	-0.047	0.037	0.001	0.057	0.105	0.035	0.037	-0.069	2

Table S3.4. **Models with ΔAIC < 2 from top model for all-subsets selection with AR-1 term for hourly summertime ODBA.** All covariates are standardised within-season to mean = 0, standard dev. = 1. A spline for hour was significant in all models, and is not shown here, nor is intercept. " Sex" represents a binomial factor, where the baseline sex is male.

Temp.	Temp.^{2}	Rainfall	Rainfall^2	Wind speed	Relative humidity	Soil temp. (10 cm)	Soil temp. (30 cm)	Soil moisture	Age	BCI	Sex	BCI:Temp.	BCI:Rainfall	BCI:Sex	ΔAIC
0.067	-0.033	-	-	-	-	-	-0.043	0.042	-0.07	0.012	-	-0.042	-	-	0
0.055	-0.035	-	-	0.019	-	-	-0.041	0.042	-0.071	0.011	-	-0.043	-	-	0.6
0.068	-0.033	0.009	-	-	-	-	-0.042	0.042	-0.07	0.011	-	-0.042	-	-	1.31
0.068	-0.033	-	-	-	-	-	-0.043	0.042	-0.07	0.01	-0.005	-0.043	-	-	1.93
0.068	-0.033	-	-	-	0.004	-	-0.043	0.043	-0.07	0.012	-	-0.042	-	-	1.9

Table S3.5. **Models with ΔAIC < 2 from top model for all-subsets selection with no accounting for autocorrelation for hourly summertime ODBA.** All covariates are standardised within-season to mean = 0, standard dev. = 1. A spline for hour was significant in all models, and is not shown here, nor is intercept. " Sex" represents a binomial factor, where the baseline sex is male.

Temp.	Temp.^{2}	Rainfall	Rainfall^2	Wind speed	Relative humidity	Soil temp. (10 cm)	Soil temp. (30 cm)	Soil moisture	Age	BCI	Sex	BCI:Temp.	BCI:Rainfall	BCI:Sex	ΔAIC
0.060	-0.034	-	-	0.018	-	-	-0.043	0.041	-0.072	-0.002	-	-0.046	-	-	0
0.072	-0.032	-	-	-	-	-	-0.044	0.042	-0.071	0	-	-0.046	-	-	0.32
0.073	-0.032	0.012	-	-	-	-	-0.043	0.042	-0.072	-0.002	-	-0.046	-	-	0.64
0.062	-0.034	0.01	-	0.016	-	-	-0.043	0.041	-0.072	-0.003	-	-0.046	-	-	0.82
0.06	-0.034	-	-	0.018	-	-	-0.044	0.041	-0.072	-0.004	-0.014	-0.047	-	-	1.54
0.058	-0.034	-	-	0.018	-0.005	-	-0.043	0.041	-0.071	-0.001	-	-0.047	-	-	1.88
0.072	-0.032	-	-	-	-	-	-0.044	0.042	-0.072	-0.003	-0.013	-0.046	-	-	1.93

Table S3.6. **Models with ΔAIC < 2 from top model for all-subsets selection on halved data for hourly summertime ODBA.** All covariates are standardised within-season to mean = 0, standard dev. = 1. A spline for hour was significant in all models, and is not shown here, nor is intercept. “ Sex” represents a binomial factor, where the baseline sex is male.

Temp.	Temp.^{2}	Rainfall	Rainfall^2	Wind speed	Relative humidity	Soil temp. (10 cm)	Soil temp. (30 cm)	Soil moisture	Age	BCI	Sex	BCI:Temp.	BCI:Rainfall	BCI:Sex	ΔAIC
0.097	-0.032	0.019	-	-	-	-	-0.042	0.045	-0.075	0.006	-	-0.051	-	-	0
0.096	-0.032	-	-	-	-	-	-0.043	0.046	-0.074	0.008	-	-0.052	-	-	0.1
0.081	-0.034	-	-	0.021	-	-	-0.042	0.045	-0.074	0.007	-	-0.052	-	-	0.44
0.085	-0.034	0.016	-	0.018	-	-	-0.041	0.045	-0.075	0.005	-	-0.051	-	-	0.84
0.094	-0.033	0.017	0.012	-	-	-	-0.042	0.044	-0.074	0.007	-	-0.051	-	-	1.01
0.098	-0.032	0.019	-	-	-	-	-0.043	0.045	-0.075	0.002	-0.01	-0.051	-	-	1.76
0.097	-0.032	0.016	-	-	-	-	-0.042	0.046	-0.074	0.006	-	-0.05	0.005	-	1.83
0.096	-0.032	0.02	-	-	-0.007	-	-0.042	0.045	-0.074	0.007	-	-0.051	-	-	1.88
0.096	-0.032	-	-	-	-	-	-0.044	0.046	-0.074	0.004	-0.009	-0.052	-	-	1.88
0.082	-0.035	0.015	0.012	0.017	-	-	-0.041	0.044	-0.074	0.006	-	-0.051	-	-	1.92

Table S3.7. **Models with ΔAIC < 2 from top model for all-subsets selection with AR-1 term for hourly autumn ODBA.** All covariates are standardised within-season to mean = 0, standard dev. = 1. A spline for hour was significant in all models, and is not shown here, nor is intercept.

Temp.	Temp.^{2}	Rainfall	Rainfall^2	Wind speed	Relative humidity	Soil temp. (10 cm)	Soil temp. (30 cm)	Soil moisture	Age	BCI	BCI:Temp.	BCI:Rainfall	ΔAIC
-	-	-	-	-	-	0.099	-	-	-	-	-	-	0
-	-	-	-	-	-	0.101	-	-	-0.075	-	-	-	0.22
-	-	-	-	-	0.054	0.105	-	-	-	-	-	-	0.28
-	-	-	-	-	0.054	0.107	-	-	-0.078	-	-	-	0.46
-	-	-	-	-	-	0.101	-	-	-0.099	-0.045	-	-	1.66
-	-	-	-	-	-	-	-	-	-	-	-	-	1.68
-	-	-	-	-	-	0.107	-0.018	-	-	-	-	-	1.83
-	-	-	-	-	-	0.106	-	0.018	-	-	-	-	1.84
-	-	-	-	-	0.054	0.107	-	-	-0.104	-0.046	-	-	1.89
0.055	-0.06	-	-	-	-	-	-	-	-	-	-	-	1.94
0.081	-	-	-	-	0.066	-	-	-	-	-	-	-	1.97
-	-	0.003	-	-	-	0.099	-	-	-	-	-	-	1.98
-	-	-	-	0.005	-	0.099	-	-	-	-	-	-	1.98
-	-	-	-	-	-	0.099	-	-	-	0.007	-	-	1.9

Table S3.8. **Models with ΔAIC < 2 from top model for all-subsets selection with no accounting for autocorrelation for hourly autumn ODBA.** All covariates are standardised within-season to mean = 0, standard dev. = 1. A spline for hour was significant in all models, and is not shown here, nor is intercept.

Temp.	Temp.^{2}	Rainfall	Rainfall^2	Wind speed	Relative humidity	Soil temp. (10 cm)	Soil temp. (30 cm)	Soil moisture	Age	BCI	BCI:Temp.	BCI:Rainfall	ΔAIC
0.083	-0.099	-	-	-	-	-	0.043	-	-0.102	-0.044	-0.049	-	0
0.082	-0.096	-	-	-	-	-	0.043	-	-	-	-	-	0.1
0.083	-0.099	-	-	-	-	-	0.043	-	-	0.009	-0.049	-	0.29
0.083	-0.096	-	-	-	-	-	0.043	-	-0.076	-	-	-	0.37
0.087	-0.09	-	-	-	-	-	-	-	-	-	-	-	0.86
0.088	-0.093	-	-	-	-	-	-	-	-0.097	-0.042	-0.049	-	0.88
0.088	-0.092	-	-	-	-	-	-	-	-	0.008	-0.049	-	1.1
0.087	-0.09	-	-	-	-	-	-	-	-0.073	-	-	-	1.19
0.101	-0.097	-	-	-0.031	-	-	-	-	-0.098	-0.043	-0.049	-	1.39
0.099	-0.093	-	-	-0.031	-	-	-	-	-	-	-	-	1.41
0.092	-0.101	-	-	-0.02	-	-	0.037	-	-0.102	-0.044	-0.049	-	1.46
0.091	-0.097	-	-	-0.02	-	-	0.037	-	-	-	-	-	1.58
0.101	-0.096	-	-	-0.031	-	-	-	-	-	0.008	-0.049	-	1.62
0.086	-0.098	-	-	-	0.014	-	0.045	-	-0.103	-0.045	-0.049	-	1.71
0.1	-0.094	-	-	-0.031	-	-	-	-	-0.073	-	-	-	1.73
0.092	-0.1	-	-	-0.02	-	-	0.037	-	-	0.009	-0.049	-	1.76
0.083	-0.097	-	-	-	-	-	0.043	-	-0.102	-0.047	-	-	1.8
0.092	-0.093	-	-	-	-	-	0.043	0.014	-0.103	-0.045	-0.049	-	1.81
0.085	-0.095	-	-	-	0.014	-	0.045	-	-	-	-	-	1.82
0.091	-0.098	-	-	-0.02	-	-	0.037	-	-0.076	-	-	-	1.85
0.091	-0.09	-	-	-	-	-	0.043	0.013	-	-	-	-	1.94
0.083	-0.099	0	-	-	-	-	0.043	-	-0.102	-0.044	-0.049	-	2

Table S3.9. **Models with ΔAIC < 2 from top model for all-subsets selection on halved data for hourly autumn ODBA.** All covariates are standardised within-season to mean = 0, standard dev. = 1. A spline for hour was significant in all models, and is not shown here, nor is intercept.

Temp.	Temp.^{2}	Rainfall	Rainfall^2	Wind speed	Relative humidity	Soil temp. (10 cm)	Soil temp. (30 cm)	Soil moisture	Age	BCI	BCI:Temp.	BCI:Rainfall	ΔAIC
0.097	-0.085	-	-	-	-	-	0.053	-	-0.091	-	-	-	0
0.103	-0.077	-	-	-	-	-	-	-	-0.087	-	-	-	0.08
-	-	-	-	-	-	0.126	-	-	-0.09	-	-	-	0.16
0.096	-0.084	-	-	-	-	-	0.052	-	-	-	-	-	0.56
0.102	-0.076	-	-	-	-	-	-	-	-	-	-	-	0.57
0.094	-0.093	-	-	-	-	-	0.056	-	-0.114	-0.039	-0.061	-	0.58
-	-	-	-	-	-	0.125	-	-	-	-	-	-	0.74
0.101	-0.084	-	-	-	-	-	-	-	-0.108	-0.037	-0.059	-	0.93
0.114	-0.08	-	-	-0.028	-	-	-	-	-0.087	-	-	-	1.49
0.097	-0.085	-	-	-	-	-	0.053	-	-0.114	-0.042	-	-	1.49
0.094	-0.091	-	-	-	-	-	0.055	-	-	0.02	-0.061	-	1.58
0.103	-0.078	-	-	-	-	-	-	-	-0.108	-0.039	-	-	1.61
-	-	-	-	-	-	0.127	-	-	-0.112	-0.04	-	-	1.67
0.1	-0.083	-	-	-	0.02	-	0.056	-	-0.092	-	-	-	1.71
-	-	-	-	-	-	0.133	-	0.022	-0.093	-	-	-	1.8
0.1	-0.083	-	-	-	-	-	-	-	-	0.02	-0.059	-	1.81
0.107	-0.076	-0.017	-	-	-	-	-	-	-0.087	-	-	-	1.87
0.103	-0.085	-	-	-0.013	-	-	0.049	-	-0.091	-	-	-	1.88
-	-	-	-	-	0.018	0.128	-	-	-0.091	-	-	-	1.92
0.099	-0.084	-0.007	-	-	-	-	0.051	-	-0.091	-	-	-	1.97
0.105	-0.076	-	-	-	0.012	-	-	-	-0.087	-	-	-	1.99
0.113	-0.079	-	-	-0.028	-	-	-	-	-	-	-	-	1.99
0.097	-0.085	-	-	-	-	-	0.053	0	-0.091	-	-	-	2

Table S3.10. **Models with ΔAIC < 2 from top model for all-subsets selection for hourly springtime Activity (binomial, 0 = inactive, 1 = active).** All covariates are standardised within-season to mean = 0, standard dev. = 1. A spline for hour was significant in all models, and is not shown here, nor is intercept. " Sex" represents a binomial factor, where the baseline sex is male.

Temp.	Temp.^{2}	Rainfall	Rainfall^2	Wind speed	Relative humidity	Soil temp. (10 cm)	Soil temp. (30 cm)	Soil moisture	Age	BCI	Sex	BCI:Temp.	BCI:Rainfall	BCI:Sex	ΔAIC
1.16	-	-	-	-0.604	-	-	-	-	-	-0.198	-0.215	-	-	-1.532	0
1.17	-	-	-	-0.612	-	-	-	-	0.313	-0.076	-0.192	-	-	-1.394	0.71
1.09	-	0.025	-0.156	-0.56	-	-	-	-	-	-0.175	-0.191	-	-	-1.566	0.95
1.165	-	-	-	-0.59	-	-	-0.125	-	-	-0.22	-0.239	-	-	-1.482	0.98
1.083	-	0	-0.148	-0.556	-	-	-	-	-	-0.104	-0.145	-	0.197	-1.591	1.09
1.187	-	-	-	-0.606	-	-	-	-0.195	-	-0.386	-0.373	-	-	-1.286	1.12
1.204	-	-	-	-0.615	-	-	-	-0.249	0.362	-0.284	-0.378	-	-	-1.077	1.26
1.122	-	-0.08	-	-0.573	-	-	-	-	-	-0.144	-0.177	-	0.187	-1.536	1.34
1.132	-	-0.059	-	-0.579	-	-	-	-	-	-0.221	-0.232	-	-	-1.503	1.44
1.175	-	-	-	-0.598	-	-	-0.132	-	0.322	-0.094	-0.216	-	-	-1.341	1.58
1.124	-	0.042	-0.173	-0.566	-	-	-	-0.247	-	-0.409	-0.388	-	-	-1.262	1.61
1.14	-	0.04	-0.175	-0.574	-	-	-	-0.301	0.378	-0.302	-0.394	-	-	-1.047	1.64
1.098	-	0.02	-0.154	-0.567	-	-	-	-	0.32	-0.055	-0.173	-	-	-1.422	1.65
1.09	-	-0.004	-0.147	-0.562	-	-	-	-	0.316	0.008	-0.132	-	0.193	-1.448	1.89
1.164	-	-	-	-0.604	-	-	-	-	-	-0.213	-0.216	-0.032	-	-1.531	1.96
1.141	-0.017	-	-	-0.604	-	-	-	-	-	-0.197	-0.213	-	-	-1.534	1.99

Table S3.11. **Models with ΔAIC < 2 from top model for all-subsets selection for hourly summertime Activity (binomial, 0 = inactive, 1 = active).** All covariates are standardised within-season to mean = 0, standard dev. = 1. A spline for hour was significant in all models, and is not shown here, nor is intercept. “ Sex” represents a binomial factor, where the baseline sex is male.

Temp.	Temp.^{2}	Rainfall	Rainfall^2	Wind speed	Relative humidity	Soil temp. (10 cm)	Soil temp. (30 cm)	Soil moisture	Age	BCI	Sex	BCI:Temp.	BCI:Rainfall	BCI:Sex	ΔAIC
-0.025	-0.221	-	-	0.222	-0.188	-	-0.255	-	-0.213	0.048	-	-0.164	-	-	0
-0.032	-0.234	-0.065	0.106	0.208	-	-	-0.268	-	-0.216	0.045	-	-0.162	-	-	0.15
-0.019	-0.22	-	-	0.202	-	-	-0.26	-	-0.22	0.026	-	-0.16	-	-	0.3
0.03	-0.221	-	-	0.209	-0.177	-	-0.238	0.099	-0.219	0.046	-	-0.144	-	-	0.32
0.039	-0.219	-	-	0.19	-	-	-0.242	0.106	-0.225	0.025	-	-0.139	-	-	0.34
0.024	-0.233	-0.065	0.103	0.196	-	-	-0.25	0.102	-0.222	0.044	-	-0.142	-	-	0.37
0.033	-0.24	-	-	0.19	-	-	-0.238	0.138	-0.261	-	-	-	-	-	0.59
-0.034	-0.233	-0.052	0.096	0.221	-0.141	-	-0.263	-	-0.212	0.059	-	-0.165	-	-	0.95
-0.021	-0.218	-	-	0.223	-0.184	-	-0.258	-	-0.22	0.022	-0.145	-0.168	-	-	0.98
-0.034	-0.226	-	-	0.225	-0.199	-	-0.255	-	-	0.096	-	-0.163	-	-	1
-0.028	-0.231	-0.064	0.106	0.21	-	-	-0.271	-	-0.223	0.019	-0.144	-0.167	-	-	1.12
0.058	-0.208	-	-	0.188	-	-	-0.243	0.115	-0.204	0.079	-0.129	-0.157	-	-0.166	1.14
0.023	-0.252	-0.055	0.098	0.195	-	-	-0.246	0.135	-0.264	-	-	-	-	-	1.14
0.045	-0.216	-	-	0.191	-	-	-0.245	0.108	-0.232	-0.002	-0.149	-0.143	-	-	1.15
-0.014	-0.216	-	-	0.204	-	-	-0.263	-	-0.227	-0.001	-0.149	-0.164	-	-	1.17
0.035	-0.239	-	-	0.191	-	-	-0.24	0.139	-0.26	-	-0.165	-	-	-	1.2
0.028	-0.241	-	-	0.206	-0.145	-	-0.236	0.134	-0.263	-	-	-	-	-	1.2
0.036	-0.217	-	-	0.21	-0.172	-	-0.242	0.101	-0.226	0.019	-0.146	-0.148	-	-	1.24
-0.041	-0.24	-0.067	0.108	0.211	-	-	-0.27	-	-	0.091	-	-0.161	-	-	1.28
0.03	-0.229	-0.064	0.102	0.197	-	-	-0.253	0.103	-0.229	0.018	-0.145	-0.147	-	-	1.29
0.049	-0.209	-	-	0.206	-0.168	-	-0.24	0.108	-0.197	0.101	-0.126	-0.161	-	-0.164	1.33
-0.012	-0.21	-	-	0.22	-0.18	-	-0.258	-	-0.192	0.103	-0.126	-0.181	-	-0.157	1.34
0.02	-0.232	-0.053	0.094	0.208	-0.13	-	-0.246	0.097	-0.218	0.057	-	-0.146	-	-	1.35
-0.03	-0.234	-0.094	0.104	0.208	-	-	-0.266	-	-0.214	0.059	-	-0.159	0.059	-	1.35
0.042	-0.221	-0.064	0.101	0.194	-	-	-0.251	0.111	-0.2	0.099	-0.126	-0.159	-	-0.165	1.35
0.015	-0.248	-	-	0.187	-	-	-0.23	0.13	-0.221	0.125	-	-	-	-	1.43
-0.006	-0.209	-	-	0.201	-	-	-0.263	-	-0.199	0.078	-0.13	-0.178	-	-0.157	1.44

Table S3.12. **Models with ΔAIC < 2 from top model for all-subsets selection for hourly autumn Activity (binomial, 0 = inactive, 1 = active).** All covariates are standardised within-season to mean = 0, standard dev. = 1. A spline for hour was significant in all models, and is not shown here, nor is intercept.

Temp.	Temp.^{2}	Rainfall	Rainfall^2	Wind speed	Relative humidity	Soil temp. (10 cm)	Soil temp. (30 cm)	Soil moisture	Age	BCI	BCI:Temp.	BCI:Rainfall	ΔAIC
-	-	-0.255	-	-	-0.254	0.785	-	0.308	-	-	-	-	0
-	-	-0.322	0.182	-	-	0.82	-	0.237	-	-	-	-	0.14
-	-	-0.301	0.157	-	-0.221	0.835	-	0.3	-	-	-	-	0.32
-	-	-0.347	0.18	-	-	0.736	-	-	-	-	-	-	0.36
-	-	-0.272	-	-	-	0.759	-	0.234	-	-	-	-	0.48
-	-	-0.254	-	-	-0.254	0.787	-	0.311	-0.39	-	-	-	0.59
-	-	-0.296	-	-	-	0.677	-	-	-	-	-	-	0.68
-	-	-0.322	0.183	-	-	0.822	-	0.239	-0.392	-	-	-	0.72
-	-	-0.301	0.157	-	-0.221	0.837	-	0.303	-0.393	-	-	-	0.9
-	-	-0.347	0.181	-	-	0.738	-	-	-0.353	-	-	-	1
-	-	-0.272	-	-	-	0.762	-	0.237	-0.387	-	-	-	1.07
-	-	-0.295	-	-	-	0.679	-	-	-0.348	-	-	-	1.33
-	-	-0.29	-	-	-0.169	0.673	-	-	-	-	-	-	1.47
0.402	-0.368	-0.284	-	-	-	-	0.411	-	-0.554	-0.455	-0.455	0.204	1.53
-	-	-0.339	0.165	-	-0.137	0.729	-	-	-	-	-	-	1.59
-	-	-0.255	-	-	-0.254	0.785	-	0.309	-0.575	-0.331	-	-	1.68
-	-	-0.277	0.174	-0.117	-	0.837	-	0.231	-	-	-	-	1.7
-	-	-0.218	-	-0.143	-	0.785	-	0.227	-	-	-	-	1.81
-	-	-0.322	0.182	-	-	0.82	-	0.237	-0.577	-0.331	-	-	1.81
-	-	-0.295	0.171	-0.132	-	0.757	-	-	-	-	-	-	1.82
-	-	-0.235	-	-0.159	-	0.709	-	-	-	-	-	-	1.86
-	-	-0.23	-	-0.069	-0.236	0.795	-	0.299	-	-	-	-	1.86
0.401	-0.367	-0.283	-	-	-	-	0.41	-	-	-0.163	-0.453	0.201	1.92
0.527	-0.372	-0.214	-	-0.254	-	-	0.356	-	-0.558	-0.452	-0.45	0.199	1.93
-	-	-0.259	-	-	-0.257	0.799	-0.028	0.305	-	-	-	-	1.97
-	-	-0.255	-	-	-0.254	0.784	-	0.308	-	-0.027	-	-	1.99

2 Supplementary Figures

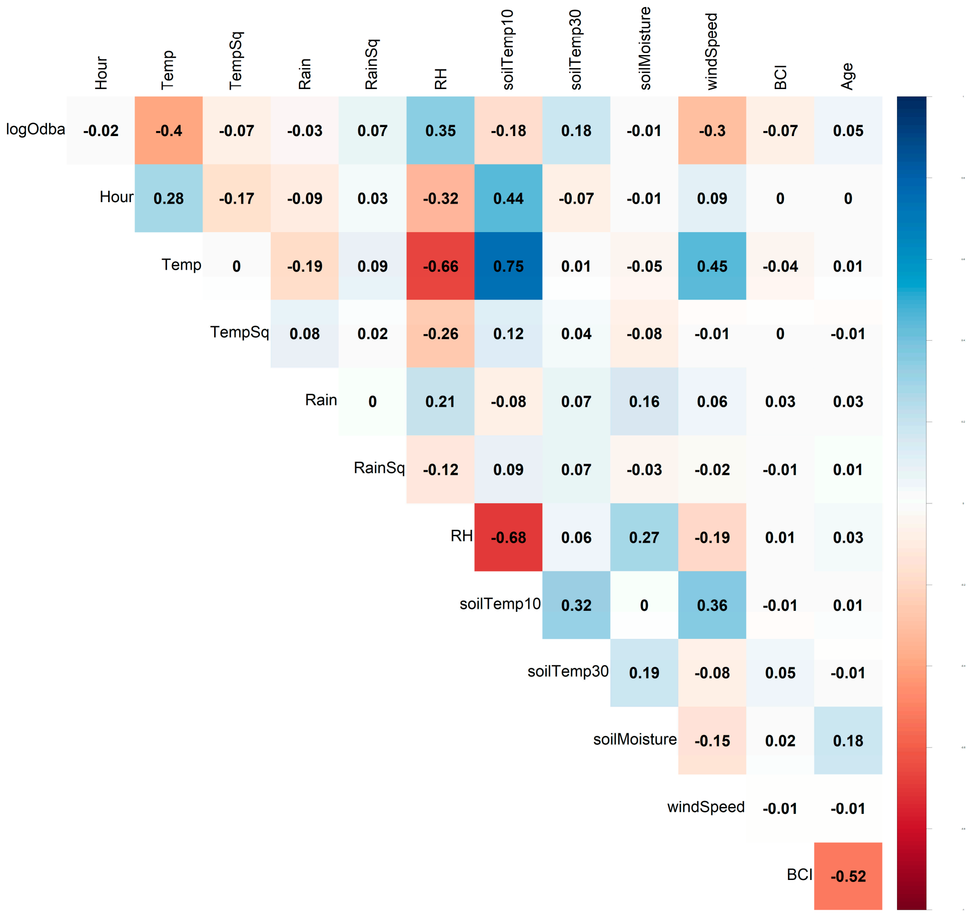

Figure S1.1 **Correlations between covariates during spring study period**. Colors denote degree of correlation, with reds indicating negative correlation and blues indicating positive correlation.

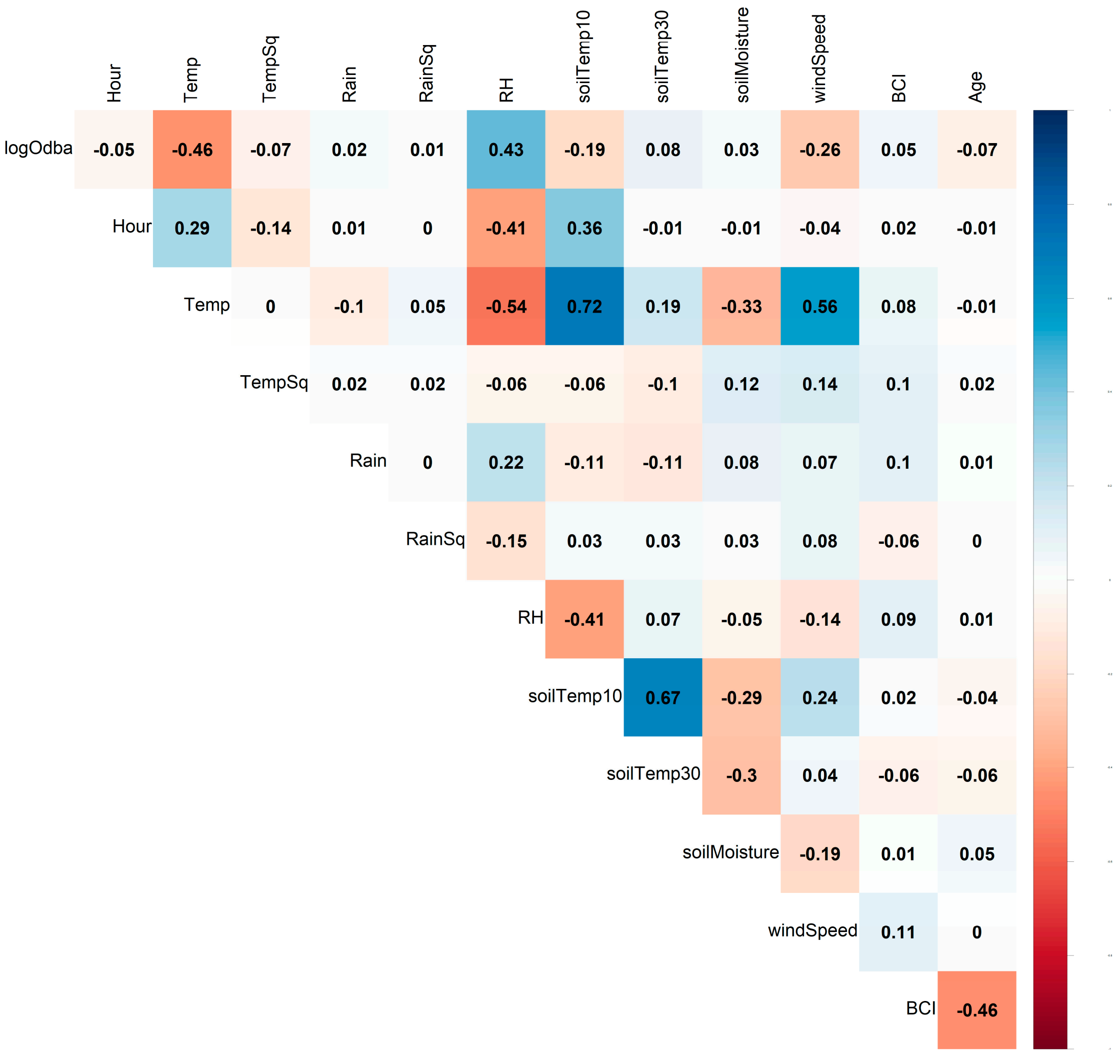

Figure S1.2 **Correlations between covariates during summer study period**. Colors denote degree of correlation, with reds indicating negative correlation and blues indicating positive correlation.

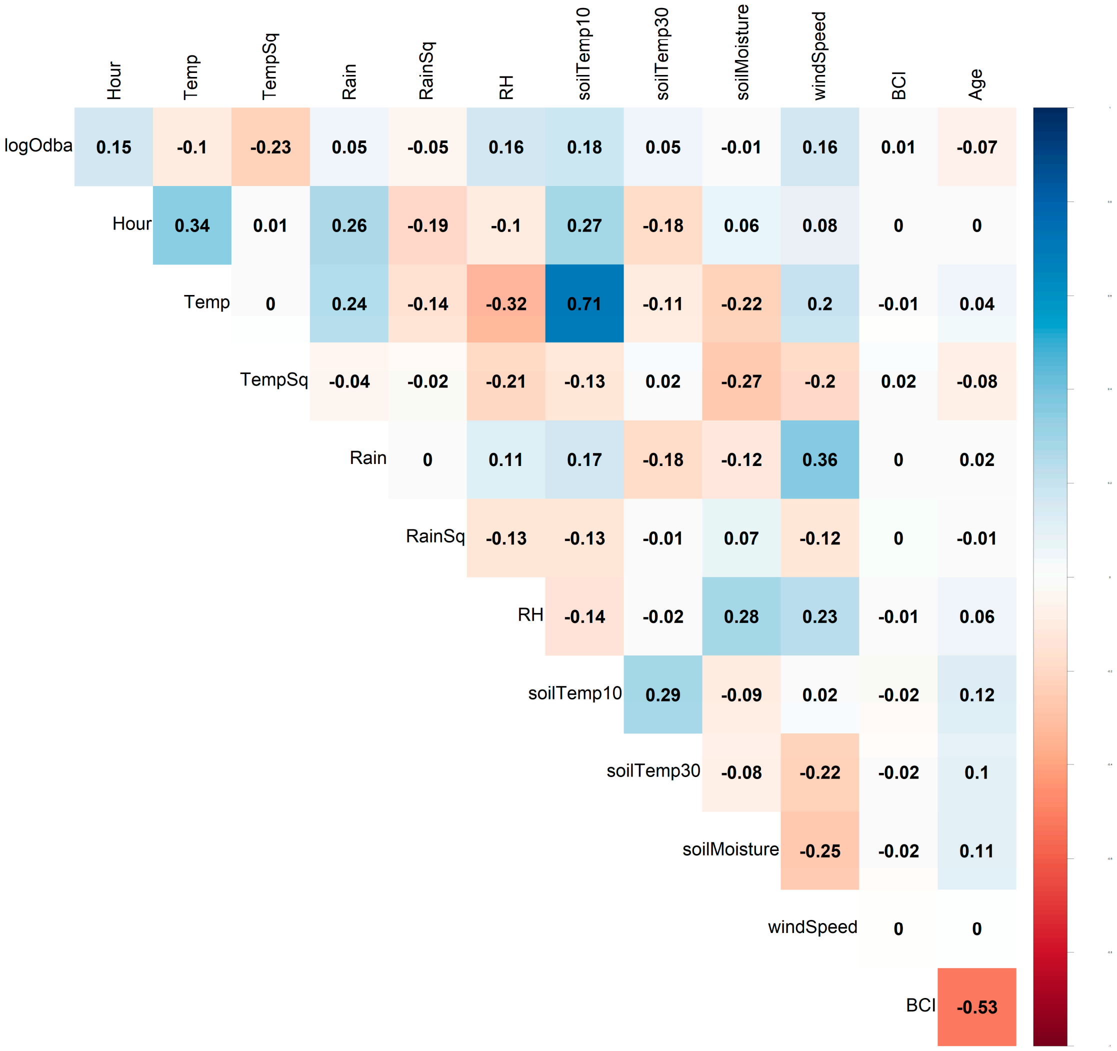

Figure S1.3 **Correlations between covariates during autumn study period**. Colors denote degree of correlation, with reds indicating negative correlation and blues indicating positive correlation.

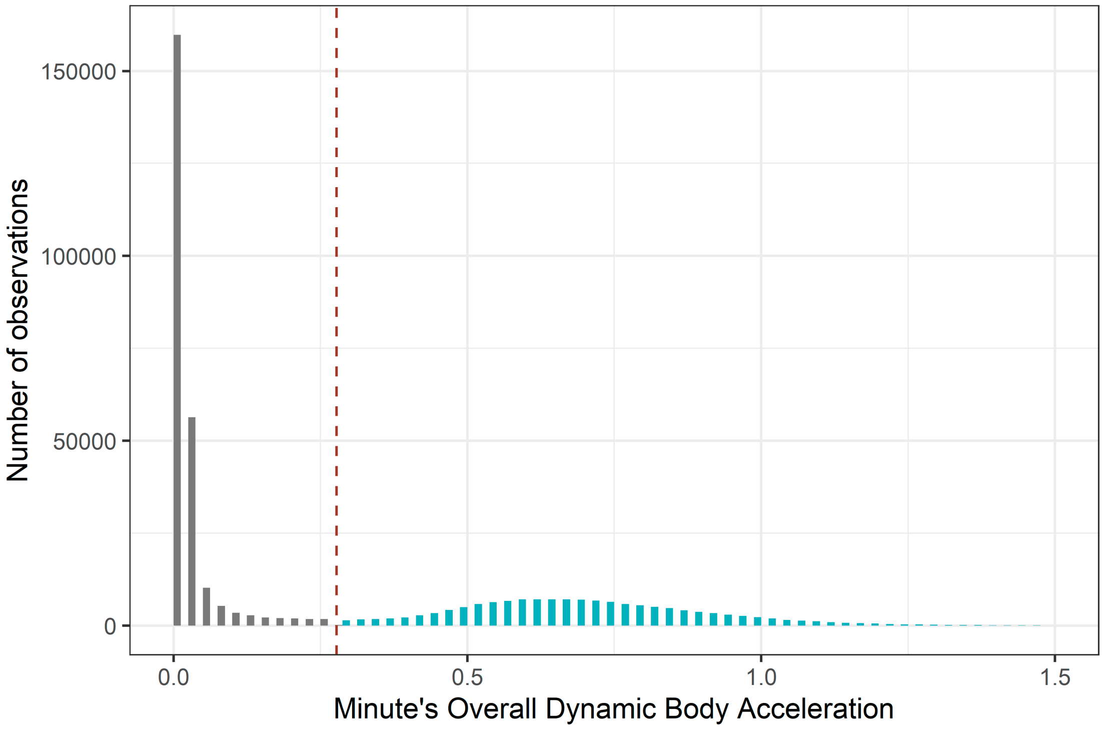

Figure S2.1 **Distribution of active/inactive samples**. Red dashed line indicates value of *T* identified by sensitivity analysis; values to the left are classified as “inactive”, to the right as “active”.
